# Identification and Expression Profiles of Chemosensory Genes in the Antennal Transcriptome of *Protaetia brevitarsis* (Coleoptera: Scarabaeidae)

**DOI:** 10.3390/insects16060607

**Published:** 2025-06-09

**Authors:** Shi-Hang Zhao, Yang Yue, Qi Gao, Rui-Tao Yu, Zhao-Hui Yang, Nan Zhou, Guo-Liang Xu

**Affiliations:** 1 Shijiazhuang Institute of Pomology, Hebei Academy of Agricultural and Forestry Sciences, Shijiazhuang 050061, China; zhaoshih3@haafs.org (S.-H.Z.); qigao977@haafs.org (Q.G.); wrightyu@haafs.org (R.-T.Y.); 2 Laboratory of Insect Collection, Shijiazhuang Institute of Pomology, Hebei Academy of Agriculture and Forestry Sciences, Shijiazhuang 050061, China; 3 College of Agriculture, Guangxi University, Nanning 530004, China; yueyang1014@163.com; 4 Agricultural Products Centre of Quality and Safety in Hebei Province, Shijiazhuang 050061, China; sjzyzh@126.com

**Keywords:** *Protaetia brevitarsis*, antennal transcriptome, chemosensory genes, tissue-specific expression

## Abstract

Chemosensory systems play a pivotal role in insect survival and reproduction by mediating the detection of volatile organic compounds in the environment. *Protaetia brevitarsis* (Coleoptera: Scarabaeidae), a phytophagous pest widely distributed across East Asia, poses a significant threat to agro-horticultural systems through crop damage. We conducted antennal transcriptome sequencing of adult beetles and identified 117 chemosensory-related genes, including 66 odorant receptors (ORs), 20 ionotropic receptors, 10 gustatory receptors, 13 odorant-binding proteins (OBPs), four chemosensory proteins, and four sensory neuron membrane proteins. Tissue-specific expression profiling revealed the antennal enrichment of five PbreOBP genes and twenty-three ORs. Notably, sexual dimorphism was observed in OR expression patterns. *PbreOR1/6/17/18/21/22/30/32* exhibited male-biased antennal expression, whereas *PbreOR25/26/29/38/41/44/61* demonstrated female-biased antennal expression, indicating their potential involvement in sex-specific behaviors, such as pheromone detection and oviposition site selection. A comprehensive description of the antenna chemosensory-related genes of *P. brevitarsis* has deepened our understanding of the olfactory mechanisms in coleopteran insects. This study also provides a basis for understanding the molecular mechanisms underlying olfaction in *P. brevitarsis*.

## Simple Summary

*Protaetia brevitarsis*, a prominent agroforestry pest widely found in East Asia, causes substantial economic losses through the direct feeding of adults on commercially valuable fruits, such as apples and grapes. Current management strategies depend predominantly on the use of chemical pesticides and physical trapping methods. However, prolonged pesticide application promotes insecticide resistance and environmental contamination, whereas conventional trapping techniques suffer from limited efficacy as they lack optimized olfactory attractants. Given the significance of the olfactory system in mediating essential behaviors, including host localization, mating, and oviposition, deciphering the chemosensory mechanisms of *P. brevitarsis* could help develop eco-friendly pest-control approaches. In this study, we comprehensively characterized the chemosensory-related gene repertoire of *P. brevitarsis* and elucidated its phylogenetic relationships and tissue-specific expression patterns. These findings provide molecular insights that will guide future functional investigations and facilitate the development of olfactory disruption-based precise pest-control technologies.

## 1. Introduction

The white-spotted flower chafer, *Protaetia brevitarsis* (Coleoptera: Scarabaeidae), is indigenous to East Asia and has established populations across most Asian countries [1,2]. *P. brevitarsis* larvae can convert organic waste into humic acid and microorganism-rich detritus, which improves disease resistance and promotes plant growth [3]. However, the adult *P. brevitarsis* is a polyphagous pest with strong dispersal capacity. It often gathers in apples, pears, grapes, and other fruits, nibbling on them and causing serious economic losses [4]. *P. brevitarsis* adults often harm fruit ripening; however, to ensure food safety, chemical pesticides cannot be used to control them. Therefore, there is an urgent need to develop environmentally friendly prevention and control strategies. Exploring the molecular mechanisms of olfactory host recognition in *P. brevitarsis* is a powerful entry point to overcome the dilemma of controlling this insect.

Olfaction saliently participates in mediating essential insect behaviors, including host recognition, mate localization, oviposition site selection, and predator avoidance [5,6,7]. Insect antennae possess a large number of olfactory sensory neurons (OSNs) that are used to perceive and recognize odor molecules in the environment, convert chemical signals into electrical signals, and transmit them to the higher nervous system in the brain, ultimately guiding the organism to make corresponding behavioral responses [8,9]. These processes require the involvement of various olfactory proteins, namely odorant-binding proteins (OBPs), chemosensory proteins (CSPs) and sensory neuron membrane proteins (SNMPs), odorant receptors (ORs), ionotropic receptors (IRs), and gustatory receptors (GRs) [10,11,12].

In insects, OBPs and CSPs are involved in the first step of chemical signal recognition and are abundant in the lymph fluid of the antennal sensory apparatus [13]. OBPs constitute a family of small soluble proteins (100–160 amino acids) characterized by conserved cysteine residues that dictate structural classification [14,15]. Based on the cysteine number and spatial arrangement, they are categorized into five subtypes: Classical, Minus-C, Plus-C, Dimer, and Atypical OBPs [11,16]. The most common are Classical OBPs, which have six conserved cysteine residues that form three interlocking disulfide bonds, fold to form a very tight and stable hydrophobic binding cavity, and increase the stability of the OBP structure [17,18]. CSPs represent a distinct protein family with shorter sequences (100–120 amino acids) and greater evolutionary conservation [19]. Their tertiary structure features two disulfide bonds between four conserved cysteines, creating a ligand-binding channel that is essential for odorant transport [20,21]. Based on sequence similarity and intron location, SNMPs can be classified as SNMP1 and SNMP2 [22,23]. SNMP1 is specifically expressed in the antennae and is associated with pheromone-specific olfactory neurons, indicating their central role in pheromone detection [24,25]. SNMP2 is also associated with the detection of pheromones but is only expressed in sensilla support cells and not in antennal neurons [26]. Recently, a similar third type of SNMP (SNMP3) has been reported in the genome of moths [27]. In addition, a genealogy-specific extension of SNMPs has been found in the Chelonidae, which defines a new set of SNMPs (named SNMP4) [24].

The chemoreceptors of insects consist of three families of proteins, namely ORs, IRs, and GRs, which are activated to convert chemical signals into electrical signals that are transmitted to the brain to guide an insect’s behavioral response [28,29]. ORs belong to the G protein-coupled receptor (GPCR) superfamily and are composed of 400–450 amino acids, usually containing seven α-helix transmembrane domains [30]. ORs are mainly distributed in the antennae and maxillary palps of insects [31]. However, unlike GPCRs, insect ORs exhibit the opposite topology, with the N-terminus located intracellularly and the C-terminus positioned extracellularly. There is no sequence similarity between OR and GPCR [32]. ORs are odor-gated ion channels consisting of a variable odor-specific subunit and a conserved odorant receptor co-receptor (ORco) subunit, and OR and ORco are assembled in a stoichiometric ratio of 1:3 [33,34]. IRs evolved from ionotropic glutamate receptors (iGluRs), which are a conserved family of synaptic ligand-gated ion channels [35]. The IR complex consists of a selectively expressed “tuning” IR and one of two widely expressed co-receptors (IR8a or IR25a), which determine the sensory response specificity of the complex [36]. GRs mediate chemosensory function through the formation of ligand-gated cation channels and belong to the superfamily of seven transmembrane ion channels (7TMICs) with low sequence similarity [37]. GRs can detect various chemical stimuli that control insect behavior and physiology. Many GRs are expressed in gustatory receptor neurons, including carbon dioxide [38], sugar [39], and bitter taste receptors [40], which regulate insect feeding.

In the present study, transcriptome sequencing was performed on the antennae of *P. brevitarsis* using Illumina sequencing technology, and six olfactory genes were identified and analyzed. Real-time quantitative PCR (RT-qPCR) was used to analyze the tissue expression profiles of OBPs and ORs. In summary, our study not only establishes a foundation for subsequent research on olfactory mechanisms but also holds significant implications for developing green control strategies targeting the olfactory system of *Protaetia brevitarsis*.

## 2. Materials and Methods

### 2.1. Insects and Tissue Collections

Adult *P. brevitarsis* were obtained from a standardized breeding facility in Nanyang City, Henan Province, China. The insect population was reared under controlled laboratory conditions in an artificial climate chamber (26 ± 1 °C, 70 ± 5% RH, and 14:10 h L:D photoperiod) through successive generations. Fresh apple slices were provided ad libitum as the primary diet, with the daily replacement of food substrates to maintain nutritional consistency.

Antennal tissues were dissected from sexually mature males and females (50 antennae per sex). Total RNA was extracted from the pooled samples (male and female antennae processed separately) for transcriptome sequencing. Three biological replicates were included in the RNA-seq analysis to ensure statistical robustness.

For expression profiling, different tissue samples were collected from sexually mature male and female adults, including the antenna, head, thorax, abdomen, legs, and wings. All samples were flash-frozen in liquid nitrogen within 2 min post-dissection and stored at −80 °C until RNA extraction. The experimental design incorporated three biological replicates (distinct individuals) with three technical replicates per sample to control procedural variability.

### 2.2. RNA Extraction, Library Preparation, and Transcriptome Sequencing

Total RNA was isolated using TRIzol™ Reagent (Invitrogen, Carlsbad, CA, USA), following the manufacturer’s protocol. RNA integrity was verified using 1.2% agarose gel electrophoresis, whereas concentration and purity were determined spectrophotometrically (NanoDrop™ 2000; Thermo Fisher Scientific, Waltham, MA, USA) with absorbance ratios of A260/A280 > 1.8 and A260/A230 > 2.0. High-quality RNA samples (3 μg/replicate) were processed for library preparation.

RNA sequencing libraries were constructed using the TruSeq™ Stranded mRNA Library Prep Kit (Illumina, San Diego, CA, USA) following a standardized workflow. In brief, polyadenylated mRNA was isolated from total RNA through poly(dT)-immobilized magnetic bead capture as the initial step. PolyA-selected mRNA was fragmented using divalent cations in a proprietary buffer (Illumina). First-strand cDNA synthesis was performed using random hexamers with SuperScript II reverse transcriptase, followed by second-strand synthesis using DNA Polymerase I/RNase H. Blunt-end repair was achieved through exonuclease/polymerase treatment with subsequent adenylation, enabling Illumina PE adapter ligation.

Size selection (400–500 bp) was performed using AMPure XP beads (Beckman Coulter, Pasadena, CA, USA). The adapter-ligated fragments underwent 15-cycle PCR amplification using an Illumina primer cocktail. Final library validation was performed using the Agilent Bioanalyzer 2100 DNA HS assay prior to NovaSeq 6000 sequencing (Shanghai Personal Biotechnology, Shanghai, China).

### 2.3. De Novo Transcriptome Analysis and Functional Annotation

Following the basecalling conversion of raw sequencing images to nucleotide sequences, primary reads were stored in FASTA format. Initial data preprocessing involved the systematic removal of technical artifacts, including primer/adapter sequences and low-quality reads (Phred score < 20) using fastp (version 0.22.0; https://github.com/OpenGene/fastp, accessed on 9 October 2024), through quality trimming, which generated high-confidence clean reads for downstream assembly.

De novo transcriptome assembly was performed using Trinity software (version r20140413p1) with the following optimized parameters: minimum k-mer coverage (min_kmer_cov = 2) and strand-specific library type (SS_lib_type = RF), while maintaining default configurations for other parameters. Contig refinement involved two critical steps: (1) sequence identity clustering to eliminate redundant sequences and (2) hierarchical clustering of transcript isoforms using Corset (version 1.05) with default similarity thresholds. Transcriptome completeness was evaluated using BUSCO (version 5.2.2). This pipeline ultimately produced non-redundant unigenes representing distinct transcriptional units.

All unigenes underwent rigorous sequence similarity analysis using the NCBI BLASTx and BLASTn algorithms (E-value threshold < 1 × 10^−5^), with functional annotations assigned according to top-ranking alignment matches. Subsequent multilayered annotation involved (1) Gene Ontology (GO) categorization using Blast2GO (b2g4pipe_v2.5) with stringent sequence similarity criteria (E-value < 1 × 10^−6^) and (2) Kyoto Encyclopedia of Genes and Genomes (KEGG) pathway mapping using KAAS (r140224 release), employing ultrastrict alignment thresholds (E-value < 1 × 10^−10^). This tiered annotation strategy ensured the progressive refinement of functional predictions across biological databases with differential stringency requirements.

### 2.4. Identification of Chemosensory Genes

Chemosensory-related sequences were identified from the *Ophraella communa* antennal transcriptome unigene library through the screening of keywords such as odorant-binding protein, chemosensory protein, sensory neuron membrane protein, odorant receptor, ionotropic receptor, and gustatory receptor. Candidate sequences were subsequently validated via a BLASTx similarity search against the NCBI non-redundant protein database. To validate the genomic context of chemosensory genes, the protein sequences of antennal transcriptome-derived candidates (CSPs, OBPs, ORs, GRs, IRs, and SNMPs) were aligned against the *P. brevitarsis* reference genome [1] using TBtools-II (version 2.301) with BLAST (https://www.ncbi.nlm.nih.gov, accessed on 18 May 2025). High-confidence hits were defined as matches with an E-value ≤ 1 × 10^−50^.

The ORF finder (http://www.ncbi.nlm.nih.gov/gorf/gorf.html, accessed on 12 December 2024) was used to analyze the open reading frames of candidate olfactory genes, NovoPro (https://www.novopro.cn/, accessed on 13 December 2024) was used to predict the transmembrane domains of candidate OR, GR, and IR genes, and SignalP 5.0 (https://www.novopro.cn/, accessed on 13 December 2024) was used to predict the signal peptide sequences of candidate OBP, CSP, and SNMP genes.

### 2.5. Sequence and Phylogenetic Analysis

The amino acid sequences of the candidate chemosensory-related genes were aligned using ClustalW (https://www.genome.jp/tools-bin/clustalw, accessed on 20 December 2024), followed by phylogenetic tree construction using the neighbor-joining method with the p-distance model and 1000 bootstrap replicates in MEGA7 (version 7.0; https://www.megasoftware.net, accessed on 6 January 2025). The resulting trees were visualized and annotated using FigTree (version 1.4.3; https://tree.bio.ed.ac.uk/software/figtree/, accessed on 7 January 2025). Comparative analysis incorporated published Coleoptera sequences retrieved using NCBI BLASTx (https://www.ncbi.nlm.nih.gov, accessed on 6 January 2025), included 129 OR datasets (34 from *Anomala corpulenta*, 8 from *Holotrichia oblita*, 24 from *Holotrichia parallela*, and 63 from *Tribolium castaneum*), 34 IR datasets (5 from *A. corpulenta*, 20 from *H. parallela*, 8 from *T. castaneum*, and 1 from *Rhyzopertha dominica*), 34 GR datasets (7 from *A. corpulenta*, 3 from *H. oblita*, 6 from *H. parallela*, 9 from *Onthophagus taurus*, and 9 from *Pachyrhinus yasumatsui*), 17 SNMP datasets (1 from *A. corpulenta*, 1 from *Diabrotica virgifera virgifera*, 2 from *H. parallela*, 4 from *P. yasumatsui*, 3 from *Trypoxylus dichotomus*, 2 from *Pyrrhalta aenescens*, 1 from *R. dominica*, 4 from *Sitophilus zeamais*, 2 from *Tenebrio molitor*, and 4 from *T. castaneum*); 150 OBP sequence datasets (22 from *T. castaneum*, 50 from *H. oblita*, 15 from *A. corpulenta*, 25 from *H. parallela*, 4 from *Hylamorpha elegans*, and 34 from *S. zeamais*); 51 CSP sequence datasets (2 from *H. oblita*, 5 from *A. corpulenta*, 16 from *H. parallela*, 8 from *R. dominica*, and 20 from *T. castaneum*) for comparison.

### 2.6. Quantitative Real-Time PCR Analysis

Gene-specific primers for quantitative reverse transcription PCR (qRT-PCR) were designed using Premier Primer 5.0 (Premier Biosoft; https://www.premierbiosoft.com/, accessed on 16 January 2025), with GAPDH2 serving as the internal reference gene [41] (primer sequences listed in Table S1; amplification efficiencies and R^2^ values derived from 8-point standard curves are provided in Table S2). Tissue-specific cDNA templates were prepared from the antennae, heads (antennae removed), thoraxes, abdomens, legs, and wings of adult beetles. Amplification reactions were performed using the Hieff qPCR SYBR Green Master Mix (TransGen Biotech, Beijing, China) on an ABI 7500 Fast Real-Time PCR System (Thermo Fisher Scientific) under standardized cycling conditions [42]. Initial denaturation was performed at 95 °C for 5 min, 40 cycles of 95 °C for 10 s and 60 °C for 30 s, followed by melting curve analysis. Three biological replicates (each containing three technical replicates) were analyzed for each tissue type. Relative gene expression levels were calculated using the 2^−ΔΔCt^ method, with tissue-specific differences assessed using one-way ANOVA (*p* < 0.05 significance threshold) [43].

## 3. Results

### 3.1. Overview of the Transcriptome

The antennal transcriptomes of female and male adults were sequenced using Illumina-based second-generation sequencing. De novo assembly was performed using Trinity with the default parameters to generate 120,436 transcripts. Subsequent redundancy reduction using CD-HIT yielded 50,162 total unigenes. The Busco analysis confirmed the completeness of the transcriptome (C: 91.2% [S: 87.0%; D: 4.2%]) (Figure S1). The unigene set had a total length of 48,420,970 bp, with an average length of 965 bp (N50 = 1413 bp; N90 = 405 bp), and GC content of 40.27% (Table 1).

### 3.2. BLAST Analysis

**Figure 1:**
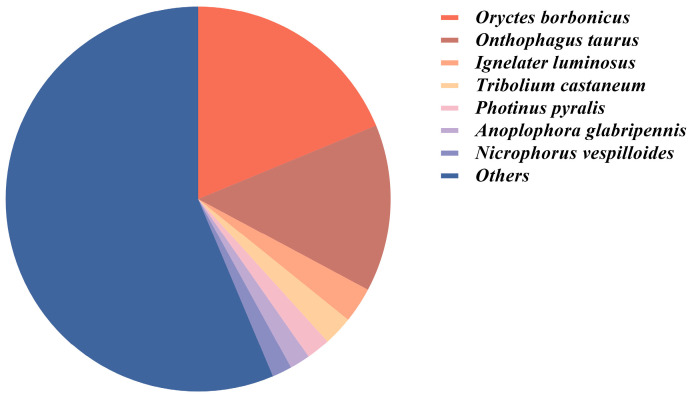
Proportion of unigene annotated species in the antennal transcriptome of *P. brevitarsis*.

BLAST similarity analysis against the NCBI non-redundant (nr) database identified significant matches for 24,833 unigenes (49.51% of the total). Taxonomic distribution revealed the strongest sequence similarity with *Oryctes borbonicus* (18.78%), followed by *Oryctes taurus* (14.03%), *Ignelater luminosus* (2.98%), *Tribolium castaneum* (2.48%), and *Photinus pyralis* (2.01%) (Figure 1). BLAST analysis identified 21 chemosensory genes (e.g., PbreOBP3, PbreCSP2, PbreIR3) with 100% identity to reference sequences, characterized by near-zero E-values and high BitScores, confirming their precise genomic localization and evolutionary conservation. Notably, PbreCSP2 (119 aa, E-value = 1.53 × 10^−85^) and PbreIR5 (379 aa, E-value = 0, BitScore = 765) exhibited complete sequence integrity. Genes with ≥95% identity suggested minor polymorphisms. Moderately conserved genes (70–95% identity, e.g., PbreOR21: 94.25% identity) indicated potential functional diversification (Table S3).

**Figure 2:**
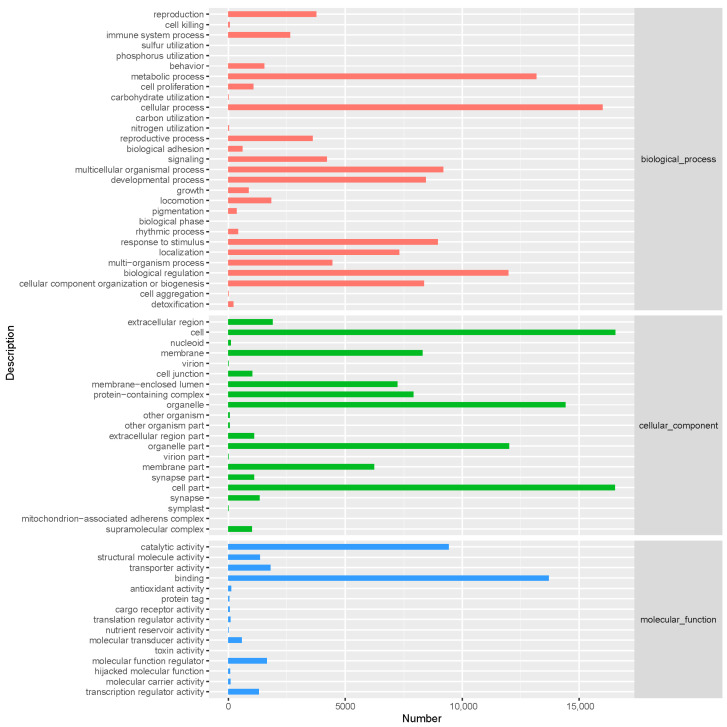
Gene Ontology (GO) classification of *P. brevitarsis*.

Gene Ontology (GO) analysis successfully annotated 18,666 unigenes (37.21% of the total) and classified them into three functional domains: biological processes (BPs), cellular components (CCs), and molecular functions (MFs). The dominant categories were cellular processes in the BPs (16,001 unigenes), cells in the CCs (16,546 unigenes), and binding in the MFs (13,707 unigenes) (Figure 2).

**Figure 3:**
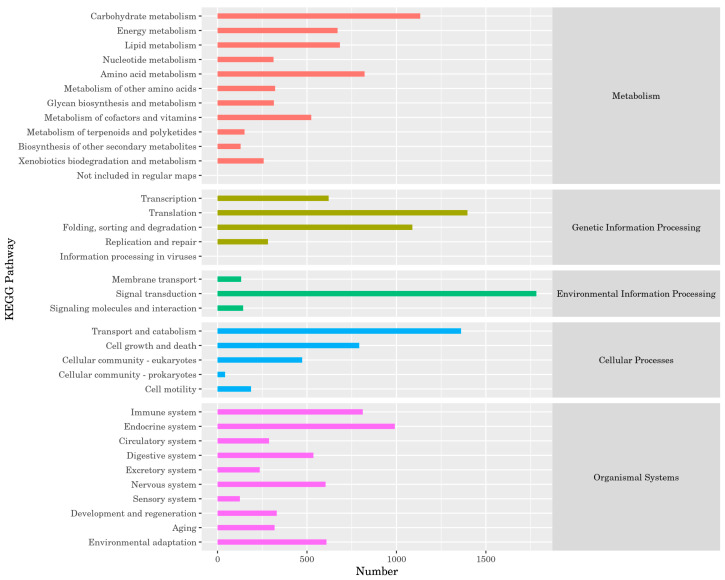
Kyoto Encyclopedia of Genes and Genomes (KEGG) classification of *P. brevitarsis*.

According to the Kyoto Encyclopedia of Genes and Genomes (KEGG) results, the proportion of unigenes involved in carbohydrate metabolism was the highest (1133 unigenes). During genetic information processing, the number of unigenes involved in folding, sorting, and degradation was the largest (1397). In environmental information processing, the largest number of unigenes participated in signal transduction (1782). Among the cellular processes, the number of unigenes involved in transport and catabolism was the largest (1361). Among the organismal systems, the largest number of unigenes belonged to the endocrine system (991) (Figure 3).

### 3.3. Identification of Candidate OBP Genes

**Figure 4:**
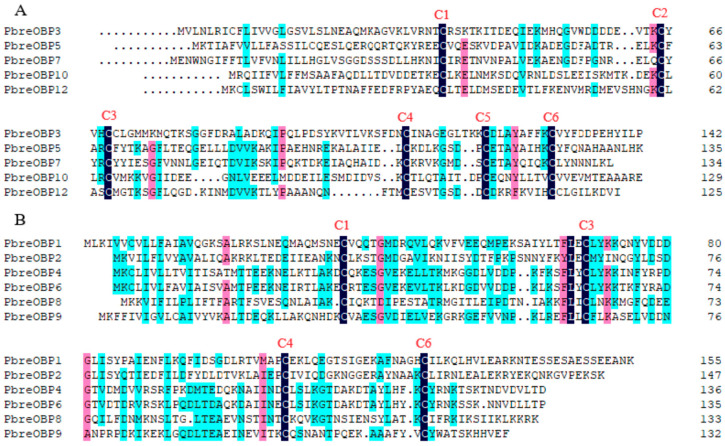
Multiple sequence alignment of odorant-binding proteins (OBPs) in *P. brevitarsis*. (**A**) Classic OBP subfamily showing six conserved cysteine residues (C1–C6). (**B**) Minus-C OBP subfamily lacking C2 and C5 residues. Conserved cysteine residues are labeled C1–C6.

**Figure 5:**
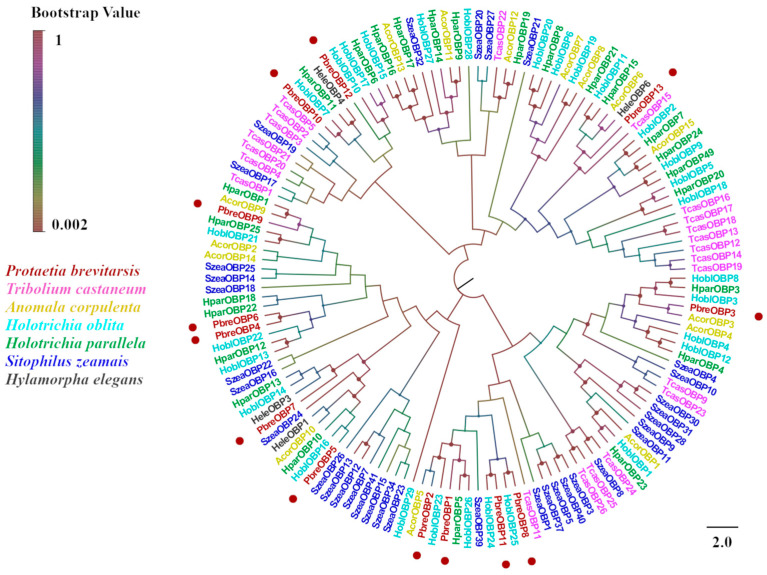
Phylogenetic tree of *P. brevitarsis* OBP genes. In total, 150 OBP sequence datasets (22 from *T. castaneum*, 50 from *H. oblita*, 15 from *A. corpulenta*, 25 from *H. parallela*, 4 from *H. elegans*, and 34 from *S. zeamais*) for comparison.

Thirteen putative OBPs designated PbreOBP1–PbreOBP13 were identified in *P. brevitarsis*. Among these, 11 OBPs contained complete open reading frames (ORFs) encoding 125–156-amino-acid-long polypeptides with predicted signal peptides. Phylogenetic classification divided the identified OBPs into two evolutionary subfamilies: Classic OBPs (five members) characterized by six conserved cysteine residues and Minus-C OBPs (six members) lacking C2 and C5 residues (Figure 4). BLASTx analysis revealed 32–85% amino acid identity with Coleopteran, with PbreOBP11 exhibiting exceptional similarity (85%) to *H. parallela* odorant-binding protein 27 (ALP75940.1) (Table S4). Phylogenetic analysis showed that PbreOBPs were divided into different clades, with PbreOBP4 and PbreOBP6 clustered together and most closely related. PbreOBPs clumped together with the OBPs of other Coleopteran insects and clustered closely in multiple clades in *H. oblita* and *A. corpulenta* OBPs (Figure 5).

### 3.4. Identification of Candidate CSP Genes

**Figure 6:**
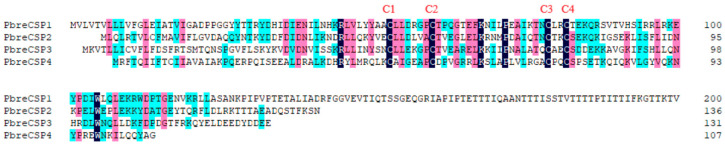
Multiple sequence alignment of chemosensory proteins (CSPs) in *P. brevitarsis*. Conserved cysteine residues are marked with a red letter (C1–C4).

**Figure 7:**
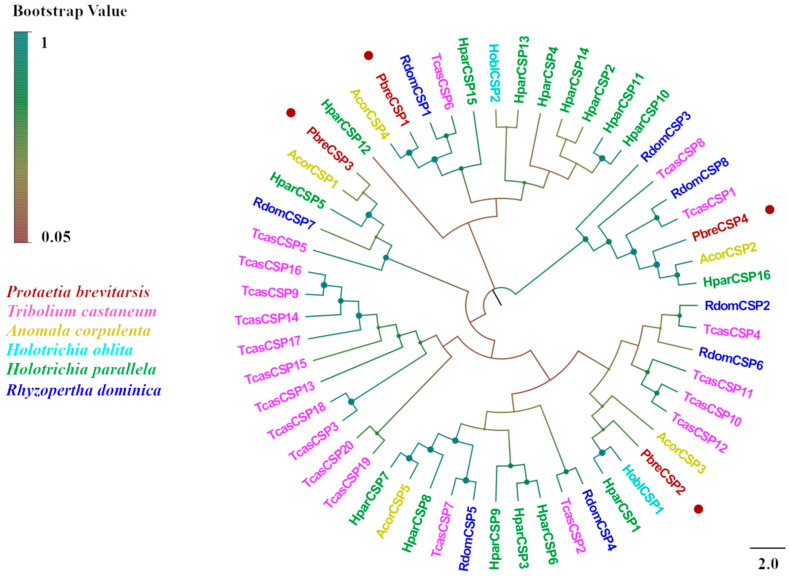
Phylogenetic tree of *P. brevitarsis* CSP genes. In total, 51 CSP sequence datasets (2 from *H. oblita*, 5 from *A. corpulenta*, 16 from *H. parallela*, 8 from *R. dominica*, and 20 from *T. castaneum*) for comparison.

We identified four candidate CSPs from the antennal transcriptome analysis of *P. brevitarsis* and named them PbreCSP1–PbreCSP4 (Table S4). Sequence analysis showed that all putative CSPs had complete ORFs and signal peptides encoding 107–241 amino acids. Upon further multiple sequence comparisons, all four CSPs were found to have four highly conserved cysteine sites (C1–C4) that are hallmarks of insect chemosensory proteins (Figure 6). Coleopteran CSPs showed stronger phylogenetic conservation compared to OBPs and ORs. The amino acid sequence similarity between PbreCSPs and Coleoptera proteins was 70–91% according to BLASTx comparison. Phylogenetic analysis revealed that all PbreCSPs formed a monophyletic clade with Coleopteran CSPs and clustered closely with AcorCSPs, with high bootstrap support values at all branch nodes (Figure 7).

### 3.5. Identification of Candidate SNMP Genes

**Figure 8:**
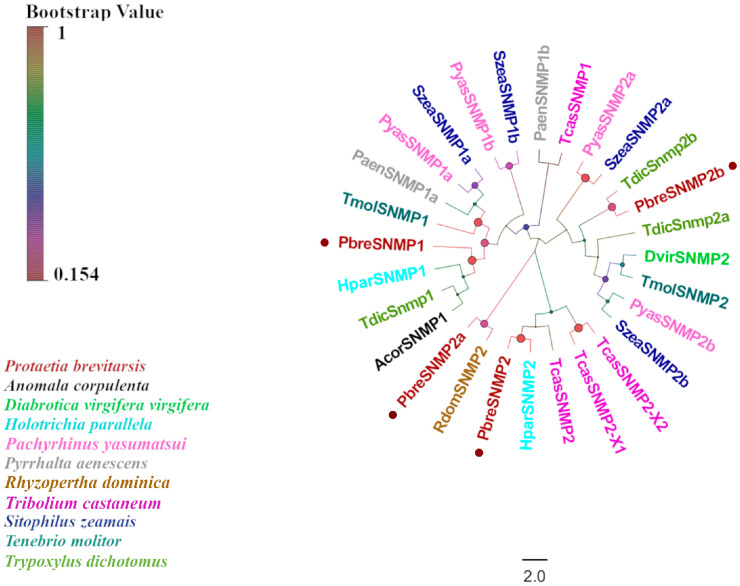
Phylogenetic tree of *P. brevitarsis* SNMP genes. In total, seventeen SNMP datasets for comparison (one from *A. corpulenta*, one from *D. virgifera virgifera*, two from *H.parallela*, four from *P.yasumatsui*, two from *P. aenescens*, one from *R. dominica*, four from *S. zeamais*, two from *T. molitor*, four from *T. castaneum*, and three from *Trypoxylus dichotomus*).

Through the antennal transcriptome analysis of *P. brevitarsis*, we identified four candidate genes encoding SNMPs, designated as PbreSNMP1–PbreSNMP4 (Table S5). Sequence characterization revealed that PbreSNMP1 and PbreSNMP2 possessed complete ORFs encoding 547 and 571 amino acid residues, respectively. Transmembrane domain prediction revealed three transmembrane domains (TMDs) in PbreSNMP1, whereas PbreSNMP2 contained two TMDs. BLASTx alignment demonstrated that all candidate SNMPs shared over 50% amino acid sequence similarity with Coleopteran proteins. Phylogenetic analysis further showed that PbreSNMP1 and PbreSNMP2 formed a highly supported monophyletic clade with other Coleopteran SNMPs, whereas PbreSNMP3 and PbreSNMP4 exhibited substantial genetic divergence in the evolutionary tree (Figure 8).

### 3.6. Identification of Candidate OR Genes

**Figure 9:**
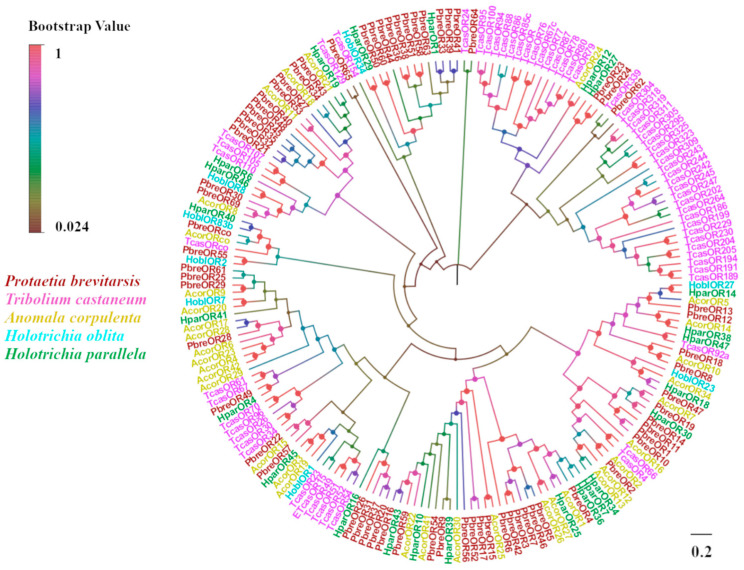
Phylogenetic tree of *P. brevitarsis* OR genes. In total, 129 OR datasets for comparison (34 from *A. corpulenta*, 8 from *Holotrichia oblita*, 24 from *Holotrichia parallela*, and 63 from *T. castaneum*).

The transcriptomic analysis of *P. brevitarsis* antennae identified 66 candidate odorant receptors (ORs), designated as PbreOR1–PbreOR66 (Table S5). A non-canonical odorant receptor co-receptor (PbreORco) exhibited conserved insect OR features, including a complete ORF encoding a seven-TMD protein. Twelve PbreORs had seven TMDs with protein sequences of more than 360 amino acids. Seven PbreORs were highly differentiated (OR21, OR31, OR37, OR43, OR61, OR62, and OR63) with <50% OR similarity to other Coleopterans. The phylogenetic tree showed that PbreORs clustered into different branches. PbreORco aligned with AcorORco, HoblORco, and TcasORco to form a separate branch of ORco (Figure 9).

### 3.7. Identification of Candidate IR Genes

**Figure 10:**
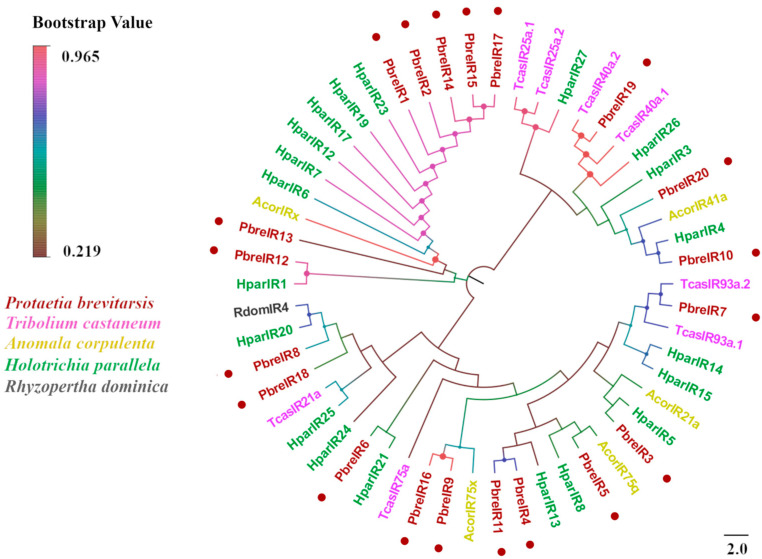
Phylogenetic tree of *P. brevitarsis* IR genes. In total, 34 IR datasets for comparison (5 from *A. corpulenta*, 20 from *H. parallela*, 8 from *T. castaneum*, and 1 from *R. dominica*).

An antennal transcriptome analysis of *P. brevitarsis* identified 20 candidate IRs (Table S5). Sixteen IRs contained complete ORFs encoding proteins ranging from 126 to 829 residues, whereas four truncated variants (PbreIR13, IR15, IR18, and IR19) exhibited 3′-terminal truncations (143–291 residues). Transmembrane domain prediction identified 0–4 TMDs per receptor, with four PbreIRs containing 4 TMDs and six possessing 3. BLASTx analysis showed that the amino acid sequence identity of all candidate IRs with other Coleopteran proteins was >50%. Phylogenetic analysis demonstrated that PbreIR5 clustered within the 75q clade alongside AcorIR75q, whereas other PbreIRs were distributed across multiple evolutionary branches (Figure 10).

### 3.8. Identification of Candidate GR Genes

**Figure 11:**
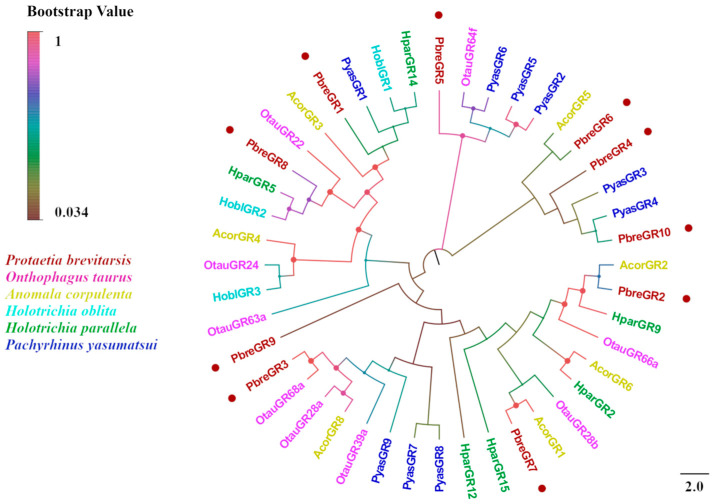
Phylogenetic tree of *P. brevitarsis* GR genes. In total, thirty-four GR datasets for comparison (seven from *A. corpulenta*, three from *H. oblita*, six from *H. parallela*, nine from *O. taurus*, and nine from *P. yasumatsui*).

Ten candidate GRs were identified in the antennal transcriptome of *P. brevitarsis*. Sequence analysis revealed that eight PbreGRs possessed complete ORFs encoding polypeptides of 100–441 residues, whereas the remaining two (PbreGR4 and PbreGR6) exhibited 3′-terminal truncations encoding truncated proteins of 236 and 320 residues, respectively (Table S5). Transmembrane domain prediction indicated that the candidate GRs contained 0–7 TMDs. BLASTx analysis revealed that the amino acid sequence identity of all GR candidate genes with other Coleopteran proteins was >50%, indicating that GRs are highly conserved in Coleopterans. Phylogenetic reconstruction demonstrated that PbreGRs are distributed across multiple subfamilies: PbreGR1 clustered within the carbon dioxide receptor clade, PbreGR5 phylogenetically aligned with the sugar receptor clade, and PbreGR4/6/10 formed a monophyletic group with the fructose receptor clade (Figure 11).

### 3.9. Tissue-Specific Expression Profiling of the Candidates Chemosensory Genes

**Figure 12:**
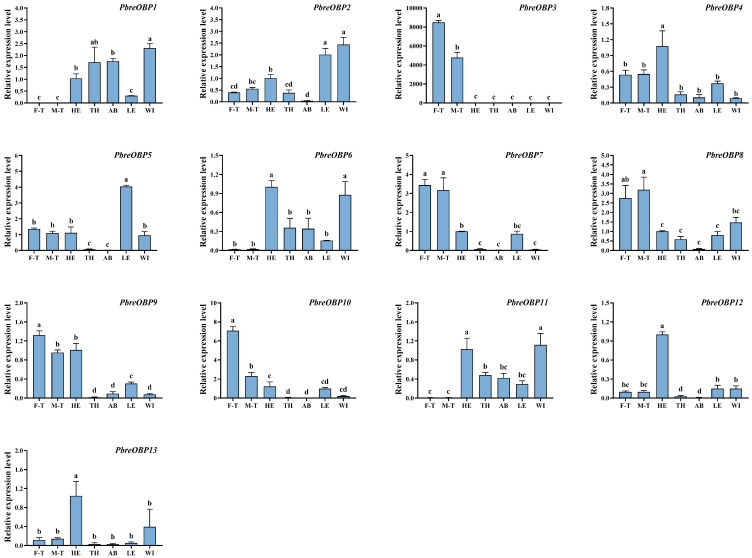
Tissue-specific expression profiles of *PbreOBP* genes in *P. brevitarsis* adults. Tissue abbreviations: F-T, female tentacles; M-T, male tentacles; HE, heads (antennae removed); TH, thorax; AB, abdomen; LE, legs; WI, wings. Different lowercase letters indicate significant differences (*p* < 0.05).

**Figure 13:**
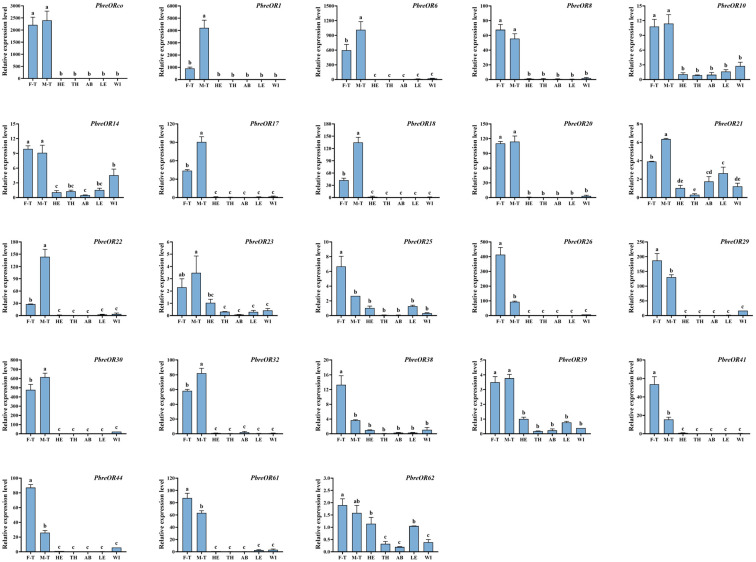
Tissue-specific expression profiles of *PbreOR* genes in *P. brevitarsis* adults. Tissue abbreviations: F-T, female tentacles; M-T, male tentacles; HE, heads (antennae removed); TH, thorax; AB, abdomen; LE, legs; WI, wings. Different lowercase letters indicate significant differences (*p* < 0.05).

To elucidate the tissue-specific and sexually dimorphic expression patterns of chemosensory-related genes in *P. brevitarsis*, we conducted an RT-qPCR analysis of 13 *PbreOBP* and 23 *PbreOR* genes with FPKM values >3 (Table S5). The results showed that *PbreOBP3/7/8/9/10* were highly expressed in the antennae. *PbreOBP2* was highly expressed in both the legs and wings. The expression of *PbreOBP4/12/13* in the head was significantly higher than that in other tissues. *PbreOBP5* exhibited leg-specific expression, whereas *PbreOBP6* and *PbreOBP11* were highly expressed in the head and wings (Figure 12). All 23 *PbreOR* genes exhibited strict antennal specificity. The expression of *PbreOR1/6/17/18/21/22/30/32* in the male antennae was significantly higher than that in the female antennae. *PbreOR25/26/29/38/41/44/61* showed biased expression in the female antennae (Figure 13).

## 4. Discussion

At present, more than 20 types of olfactory genes have been identified in Coleopteran insects, most of which belong to Cerambycidae. The olfactory genes have been reported in only eight species of Scarabaeidae, including *H. oblita* [44], *Holotrichia plumbea* [45], *P. brevitarsis* [41], *H*. *parallela* [46,47], *H. elegans* [48], *Brachysternus prasinus* [48], *O. taurus* [49], and *A. corpulenta* [50]. While prior studies in *P. brevitarsis* focused predominantly on chemosensory receptors such as ORs and GRs [41], the systematic identification of key olfactory protein families, including CSPs, OBPs, and SNMPs, remains unexplored in this species. We generated the antennal transcriptomes of adult *P. brevitarsis* males and females using Illumina next-generation sequencing technology. The antennal transcriptomes of male and female *P. brevitarsis* were generated using Illumina sequencing, which identified 117 chemosensory-related genes encompassing OBPs, CSPs, SNMPs, ORs, GRs, and IRs, all validated through stringent BLAST criteria (E-value ≤ 1 × 10^−50^) against the *P. brevitarsis* reference genome [1]. The tissue expression patterns of the key genes were validated using RT-qPCR. These findings provide crucial molecular data for deciphering the chemical communication mechanisms in Scarabaeidae and provide a theoretical foundation for developing novel pest management strategies targeting olfactory modulation.

Chemosensory systems are crucial for the survival and reproductive behavior of *P. brevitarsis*, where OBPs and CSPs mediate the initial recognition and specific binding of semiochemicals [51]. Transcriptomic analysis identified thirteen OBP and four CSP genes in *P. brevitarsis*. The number of OBPs is comparatively lower than that of other coleopterans, *Anoplophora glabripennis* (42 OBPs) [52], *A. corpulenta* (15) [50], *Plagiodera versicolora* (24) [53], and *Pachyrhinus yasumatsui* (41) [54]. In addition, the number of CSPs was similar to that of *A. corpulenta* (five CSPs) [50], *Anthonomus eugenii* (six) [55], and *Dendroctonus valens* (six) [56] but lower than *A. glabripennis* (twelve) [52] and *P. versicolora* (ten) [53]. The differential expansion of OBP/CSP gene families across insect taxa may reflect both ecological adaptation strategies and tissue-specific expression limitations. Studies have shown that some insect OBPs and CSPs are also expressed in the larval stage or other tissues; therefore, the number of genes obtained from the antennal transcriptome may be low [57]. However, cross-study comparisons require caution due to potential methodological variations in gene family annotation.

BLASTx analysis revealed evolutionary conservation between the PbreOBP/PbreCSP gene families and coleopteran chemosensory systems [58]. In addition, the sequence identity of PbreCSPs from other Coleoptera species was higher than that of PbreOBPs, perhaps because CSPs are more conserved than OBPs [59] (Table S4). The phylogenetic tree showed that OBPs and CSPs clustered together with the chemosensory genes of other Coleoptera species, suggesting that they may have similar odor recognition functions [50].

OBP expression in different tissues may indicate different physiological functions [60]. High antennal expression was observed for *PbreOBP3*, *PbreOBP7*, *PbreOBP9*, and *PbreOBP10*, indicating their potential involvement in semiochemical perception [15]. Notably, *PbreOBP9/10*, which are highly expressed in male antennae, may play an important role in mate-finding and mating behaviors [61]. Notably, *PbreOBP5* was highly expressed in the legs of the beetle, and *PbreOBP12* was specifically expressed in the head, evincing that they may have functions other than chemical communication [15]; functional validation (e.g., ligand-binding assays or RNAi) is required to clarify their specific biological significance [62]. Additionally, Coleopteran OBPs are recognized to serve non-chemosensory functions, such as participation in immune defense [63,64].

SNMPs serve as critical components in insect chemosensory systems. Their molecular functions were initially characterized in pheromone-sensitive neurons of Lepidoptera and are thought to be involved in pheromone recognition [65]. Notably, the lineage-specific expansion of SNMPs has been identified in Scarabaeidae, defining a novel subgroup within this protein family [23,66]. Transcriptomic analysis revealed four SNMP genes in *P. brevitarsis*. Based on their phylogenetic relationships, insect SNMPs can be divided into four groups (SNMP1–SNMP4).

ORs are pivotal components of the insect olfactory system. Antennal transcriptome analysis identified 66 OR genes in *P. brevitarsis*, a gene count comparable to that of the phytophagous Coleopteran species, including *Semanotus bifasciatus* (71 ORs) [67] and *Rhynchophorus ferrugineus* (76 ORs) [68]. Evolutionary analyses showed that ORco was highly conserved, and evolutionary analyses indicated that PbreORco clustered with AcorORco, HoblORco, and TcasORco to form an ORco branch. The function of ORco has been studied in various insects and is thought to be involved in mating, laying, and feeding [33,69,70]. Tissue expression profile analysis showed that 23 ORs were specifically expressed in the antennae, suggesting their important role in chemical perception. Sexual dimorphism analysis identified differential expression patterns: eight ORs (*PbreOR1/6/17/18/21/22/30/32*) displayed male-biased antennal expression, potentially mediating sex pheromone detection. Conversely, seven ORs (*PbreOR25/26/29/38/41/44/61*) exhibited female-biased antennal expression, which may be associated with oviposition-related chemosensation.

Twenty IRs were identified in *P. brevitarsis*, which exceeds the number reported in other Coleopteran species, such as *D. valens* (three IRs) [56], *P. versicolora* (seven) [53], *A. glabripennis* (four) [52], and *A. corpulenta* (five) [50], but fewer than those of *H. parallela* (twenty-seven) [47]. In addition, we analyzed and obtained ten GRs for *P. brevitarsis*, exceeding the numbers reported for *A. corpulenta* (eight GRs) [50] and *D. valens* (four) [56], but less than *O. communa* (13) [59]. At present, studies on the IRs and GRs of Scarabaeidae are limited. Early studies have reported that insect IRs are receptors for volatile substances (e.g., acids and amines) and that IRs are also capable of perceiving other odorants and are involved in non-olfactory functions, such as taste, temperature, and humidity [71]. In addition to the typical olfactory receptors, some taste receptors expressed in the olfactory organs of insects may be involved in olfactory perception, including sugar, bitter taste, and pheromone recognition receptors [72]. Phylogenetic analyses indicated that PbreGR1 clustered in the carbon dioxide receptor clade, PbreGR5 clustered in the sugar receptor clade, and PbreGR4/6/10 clustered in the fructose receptor clade. They may have a role in mediating sugar perception [73]. Currently, the research on taste receptors in the family Scarabaeidae is mainly based on genomic analysis, and to the best of our knowledge, any study on the antennal GRs in Scarabaeidae has not been reported so far. The specific functions of these genes need to be explored in the future.

While this study provides the antennal transcriptome of *P. brevitarsis*, two key limitations should be noted. First, the limited functional annotation rate (49.5%) reflects the scarcity of Scarabaeidae-specific entries in public databases (e.g., NCBI nr), particularly for lineage-specific chemosensory genes. Future studies integrating proteomic or genome-guided annotation may resolve these ‘dark’ sequences. Second, the lack of functional validation necessitates caution in interpreting gene–phenotype relationships.

## 5. Conclusions

A transcriptomic analysis of *P. brevitarsis* identified 117 chemosensory-related genes, including 13 odorant-binding proteins (OBPs), 4 chemosensory proteins (CSPs), four sensory neuron membrane proteins (SNMPs), 66 odorant receptors (ORs), 10 gustatory receptors (GRs), and 20 ionotropic receptors (IRs). Expression profile analysis revealed that *PbreOBP3/7/8/9/10* had the highest expression in the antennae, and 23 *PbreOR* genes were specifically expressed in the antennae. Subsequently, we will conduct the functional characterization of chemosensory genes through RNAi and heterologous expression combined with electroantennography and behavioral assays.

## Figures and Tables

**Table 1:** Overall statistics of antennal transcriptome of *P. brevitarsis*.

Sample Name	Transcript	Unigene
Sequence Number	120,436	50,162
Total Length (bp)	140,250,123	48,420,970
Max. Length (bp)	15,964	15,964
Mean Length (bp)	1164.52	965
SD (bp)	-	985
N50 (bp)	1732	1413
N50 Sequence No.	23,673	9434
N90 (bp)	485	405
N90 Sequence No.	83,611	36,236
GC%	39.02	40.27

## Data Availability

The data presented in this study are available in the Supplementary Materials. The sequencing data generated from this project were submitted to the NCBI SRA database linked to BioProject PRJNA1254102.

## Supplementary Materials

The following supporting information can be downloaded at: https://www.mdpi.com/article/10.3390/insects16060607/s1, Figure S1: BUSCO assessment results; Table S1: Primers for RTqPCR of PbreOBP and PbreOR genes in *P. brevitarsis*; Table S2. Primer information; Table S3. Genomic mapping validation of chemosensory genes through BLAST analysis; Table S4: The BLASTx match of *P. brevitarsis* candidate CSP and OBP genes; Table S5: The BLASTx match of *P. brevitarsis* candidate OR, IR, GR and SNMP genes; File S1: The amino acid sequences of *P. brevitarsis* putative chemosensory receptor genes.

## Abbreviations

The following abbreviations are used in this manuscript:

OSNs	Olfactory sensory neurons
OBPs	Odorant-binding proteins
CSPs	Chemosensory proteins
SNMPs	Sensory neuron membrane proteins
ORs	Odorant receptors
IRs	Ionotropic receptors
GRs	Gustatory receptors
GPCR	G protein-coupled receptor
ORco	Odorant receptor co-receptor
RT-qPCR	Real-time quantitative PCR
ORFs	Open reading frames
TMD	Transmembrane domain
