# Supplementary material for: Identification and Expression Profiles of Chemosensory Genes in the Antennal Transcriptome of Protaetia brevitarsis (Coleoptera: Scarabaeidae)
Source: Insects. 2025 Jun 9;16(6):607. doi: 10.3390/insects16060607 (PMC12193510; doi:10.3390/insects16060607)
Supplement: Supplementary file 1 [file insects-16-00607-s001.zip › insects-3634012-supplementary.pdf]

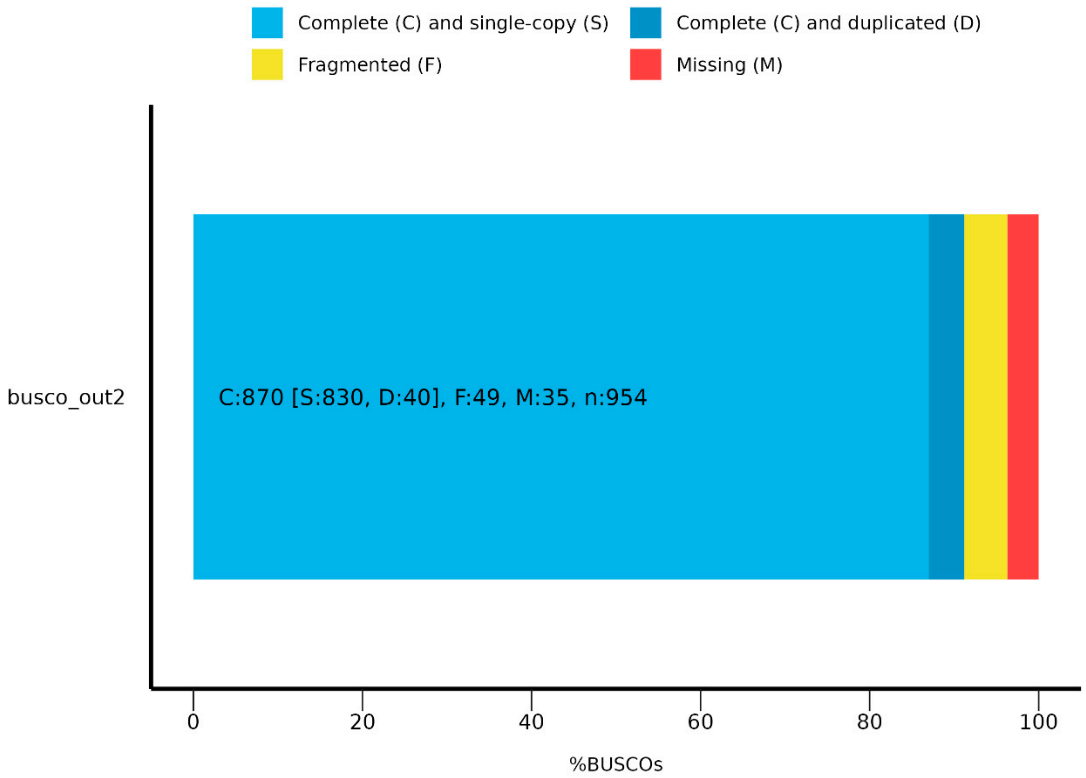

Figure S1. BUSCO Assessment Results

Table S1. Primers for RTqPCR of PbreOBP and PbreOR genes in *P. brevitarsis*.

GAPDH2F	GGCTTTTCGAGACGAACCGT	OR14F	ATAACGAGGGACCCAAAAAC
GAPDH2R	GCGGCTAAAGCTGTCGGAAA	OR14R	TAACCGAAGTCAACCATACAAA
OBP1F	AGTGTGCGTTCTTCTTTTTGC	OR17F	ATAGATGATGTGGGCGGC
OBP1R	CGATCCATTCCAGTTTGTTGT	OR17R	TGAAATTGCTTGGAGGGG
OBP2F	GGAAATTGACCGAAGATGAAA	OR18F	CGACTGCCTAATGCTACTTTCA
OBP2R	CTGAGTCGAGGTAGCCCTG	OR18R	CCAACCAATCGCTCTCCC
OBP3F	CAAATGAAAGCAGGTGTGAAA	OR20F	TTTGGATCAATCGCGGCT
OBP3R	CATACCGAGGCAACAATGGAC	OR20R	TTTTGGCTATCGTTTGTATGTG
OBP4F	CGAAGATGAAAGGCGGAGA	OR21F	TTACATGACGAAACCCGCT
OBP4R	AAGGCAATCGTTAATGATGGC	OR21R	GCAATCCACAGAAACCAAAG
OBP5F	CTCAAGTGTTTCGCCAGATGTT	OR22F	ACAAGGAATATCACCCAAAAAC
OBP5R	TGCTTTTTCACGGTTGTGTTC	OR22R	AAGCACAATAAATTGCCACAG
OBP6F	TCGGGTGTGGAGAAAGAAG	OR23F	GCGAGAAGGTCAAATCCCA
OBP6R	ATCGGCGCGGTAGAATT	OR23R	CTCCACCAAACCGCAAAC
OBP7F	GTCTTGTGTCTGGTGGTGATTC	OR25F	CGTTGGATAGAAGTGCGTGG
OBP7R	CCTTGTCCGTCTTCTGTGGT	OR25R	GGGTGAACAGAATGGGTGAGA
OBP8F	CCACTGATCTTTACGTTTGCA	OR26F	TAATGGAGCGGGCTAAGAT
OBP8R	TTGGTGTCCGGTATTTCCA	OR26R	TGACGAGGGCAAAAACAGA
OBP9F	TGTAATTGGTGTTCTGTGTGCT	OR29F	CGAACGAAAAATCCACCAA
OBP9R	AGTTTTTCCTTTATTTTGTCTGG	OR29R	GGCATATTCCAAGGACACTG
OBP10F	TATTCTTCATGTCAGCTGCATT	OR30F	GCTGCTCCGTCATTCCAG
OBP10R	CTTGGTCATTTTCGATATTTCC	OR30R	TTTTCCTCATCTCCCTCCAC
OBP11F	AACAGAACAAATAGAAGCCGTC	OR32F	CGTCAACAGCAAAAGGATACA
OBP11R	ACAAACCAGAAAAGTTTTCACAG	OR32R	CCAAAGCAGTGAGAGGCATA
OBP12F	ACTCCAACCAACGCTTTCTTT	OR38F	TTTTGCCAGTATTATTTTGTCATC
OBP12R	TTATGGCTGACCTCCATGTCAC	OR38R	AACATCGTCTCCATTGGTTTT
OBP13F	TTCTCTTCTTATTCGCTCATCTC	OR39F	TTATGGGTTTTATTGGCGAA
OBP13R	ATCCACACTCAACTTACCATCTT	OR39R	GACCGAGACAGCAGATTGG
ORcoF	TTGGTTGATCTTTGCTTGCG	OR41F	CGTTTGACAACCAGCACACT
ORcoR	GTAGCTTCCTCGTTCCTCTGG	OR41R	CCCAACACGATCGGATATAG
OR1F	CTGAGGTGAACAGAGGCGGT	OR44F	AACGAGTTTTGCGAATTGAT
OR1R	TGGTAATGGGGACGTAGGGA	OR44R	GCCTCTGCTGGAAAGGTGTA
OR6F	ACCGTTTTTTTGCCTCTCCA	OR61F	TCGACTTAAACGTGCAAAACA
OR6R	CGCCTTATCACTGCGTCCTC	OR61R	CATCAGCGTAAAAATCCAACC
OR8F	GGTTTATGGTACGCTCTCCTT	OR62F	TGAAGGTGATTATTTATGACGGG
OR8R	AGCTTTCACTCCAGTTATGGG	OR62R	GTTACATTGATAGGGAATTTGGGT
OR10F	TATGTTTTGCTATTGGGGGA	OR10R	ACAGATTTTTGACTGCGTGC

Table S2. Primer information

	Amplification efficiency (%)	R^2
GAPDH	109.6	0.996
OBP1	108.2	0.982
OBP2	98.1	0.993
OBP3	108.7	0.999
OBP4	103	0.996
OBP5	105.2	0.998
OBP6	107.8	0.994
OBP7	109.1	0.999
OBP8	92.4	0.990
OBP9	94.1	0.991
OBP10	99.3	0.996
OBP11	101.9	0.962
OBP12	108.6	0.983
OBP13	108.8	0.990
Orco	110.0	0.997
OR1	108.0	0.995
OR6	108.5	0.995
OR8	104.0	0.990
OR10	107.9	0.977
OR14	104.0	0.985
OR17	104.7	0.991
OR18	110.7	0.991
OR20	100.0	0.996
OR21	108.4	0.996
OR22	108.1	0.990
OR23	109.3	0.984
OR25	93.4	0.983
OR26	106.9	0.987
OR29	98.2	0.960
OR30	108.1	0.974
OR32	98.2	0.984
OR38	105.1	0.954
OR39	110.9	0.997
OR41	99.0	0.957
OR44	87.0	0.967
OR61	93.6	0.950
OR62	92.9	0.990
OR63	104.0	0.988

Table S3. Genomic mapping validation of chemosensory genes through BLAST analysis

QueryID	SubjectID	Identity	AlignmentLen	Mismatch	GapOpen	QueryStart	QueryEnd	SubjectStart	SubjectEnd	E-value	BitScore	SubjectDefID
PbreOBP1	gnl|BL_ORD_ID|7003	33.333	87	58	2	1	87	1	255	5.38E-07	45	Pbre006977.1
PbreOBP3	gnl|BL_ORD_ID|3726	100	142	0	0	1	142	1	426	3.14E-97	274	Pbre003763.1
PbreOBP4	gnl|BL_ORD_ID|18968	94.118	136	8	0	1	136	1	408	1.54E-91	259	Pbre019009.1
PbreOBP5	gnl|BL_ORD_ID|18963	39.837	123	74	1	6	127	22	390	3.74E-31	106	Pbre019051.1
PbreOBP5	gnl|BL_ORD_ID|18969	31.496	127	87	0	7	133	10	390	2.12E-16	69	Pbre018992.1
PbreOBP6	gnl|BL_ORD_ID|18969	99.259	135	1	0	1	135	1	405	2.77E-96	271	Pbre018992.1
PbreOBP7	gnl|BL_ORD_ID|18963	97.015	134	4	0	1	134	1	402	1.75E-79	229	Pbre019051.1
PbreOBP8	gnl|BL_ORD_ID|18969	35.211	71	46	1	49	118	148	360	2.24E-08	48	Pbre018992.1
PbreOBP9	gnl|BL_ORD_ID|18967	98.485	132	2	0	1	132	1	396	4.93E-95	268	Pbre019038.1
PbreOBP10	gnl|BL_ORD_ID|4963	98.462	130	2	0	1	130	1	390	8.70E-89	252	Pbre004965.1
PbreOBP11	gnl|BL_ORD_ID|10560	93.75	128	8	0	1	128	1	384	5.45E-86	245	Pbre010562.1

PbreOBP 12	gnl|BL_ORD_ID| 9763	100	125	0	0	1	125	1	375	1.25E-92	261	Pbre009711.1
PbreOBP 13	gnl|BL_ORD_ID| 2273	100	112	0	0	1	112	1	336	4.95E-67	197	Pbre002285.1
PbreCSP1	gnl|BL_ORD_ID| 17428	99.567	231	1	0	11	241	31	723	2.74E-137	384	Pbre017441.1
PbreCSP2	gnl|BL_ORD_ID| 20684	100	119	0	0	18	136	73	429	1.53E-85	245	Pbre020648.1
PbreCSP3	gnl|BL_ORD_ID| 20683	99.16	119	1	0	1	119	1	357	1.71E-85	244	Pbre020640.1
PbreCSP4	gnl|BL_ORD_ID| 11318	100	107	0	0	1	107	1	321	7.65E-77	220	Pbre011304.1
PbreORco	gnl|BL_ORD_ID| 20592	99.37	476	3	0	1	476	1	1428	0	990	Pbre020590.1
PbreOR1	gnl|BL_ORD_ID| 1368	85	440	66	58	1	440	781	1926	0	634	Pbre001395.1
PbreOR2	gnl|BL_ORD_ID| 789	100	122	0	0	83	204	19	384	1.82E-86	258	Pbre000787.1
PbreOR3	gnl|BL_ORD_ID| 2523	32.154	311	211	47	33	321	1	858	8.35E-38	142	Pbre002513.1
PbreOR4	gnl|BL_ORD_ID| 2523	94.479	163	9	0	11	173	862	1350	8.32E-102	309	Pbre002513.1
PbreOR5	gnl|BL_ORD_ID| 2523	29.286	140	99	4	9	146	856	1269	1.65E-13	70	Pbre002513.1
PbreOR6	gnl|BL_ORD_ID| 5410	26.908	249	182	9	30	276	220	945	4.80E-26	108	Pbre005401.1

PbreOR7	gnl|BL_ORD_ID|2523	40	25	15	0	175	199	1361	1435	6.13E-18	24	Pbre002513.1
PbreOR8	gnl|BL_ORD_ID|6307	98.013	302	6	0	99	400	1	906	0	575	Pbre006249.1
PbreOR9	gnl|BL_ORD_ID|2526	89.645	338	35	34	23	360	1	912	0	608	Pbre002596.1
PbreOR10	gnl|BL_ORD_ID|1368	97.701	87	2	0	308	394	487	747	5.32E-43	159	Pbre001395.1
PbreOR11	gnl|BL_ORD_ID|1368	97.826	276	6	0	136	411	1900	2727	2.35E-170	499	Pbre001395.1
PbreOR12	gnl|BL_ORD_ID|17211	39.568	417	252	22	3	407	10	1230	6.16E-92	281	Pbre017215.1
PbreOR13	gnl|BL_ORD_ID|17211	99.512	410	2	0	1	410	1	1230	0	806	Pbre017215.1
PbreOR13	gnl|BL_ORD_ID|9449	30.556	72	50	0	322	393	2074	2289	7.05E-06	47	Pbre009388.1
PbreOR14	gnl|BL_ORD_ID|16039	97.468	395	10	0	1	395	1	1185	0	742	Pbre016024.1
PbreOR15	gnl|BL_ORD_ID|2530	83.071	254	43	0	20	273	685	1446	2.28E-146	435	Pbre002583.1
PbreOR16	gnl|BL_ORD_ID|2525	99.022	409	4	0	1	409	1	1227	0	779	Pbre002613.1
PbreOR17	gnl|BL_ORD_ID|2525	49.497	398	201	2	11	407	28	1218	6.26E-143	412	Pbre002613.1
PbreOR18	gnl|BL_ORD_ID|6306	99.02	408	4	0	1	408	1	1224	0	832	Pbre006273.1

PbreOR19	gnl|BL_ORD_ID|20166	40.909	176	104	4	229	404	268	783	3.76E-37	134	Pbre020162.1
PbreOR20	gnl|BL_ORD_ID|15747	92.776	263	19	0	1	263	1117	1905	1.98E-168	494	Pbre015765.1
PbreOR21	gnl|BL_ORD_ID|4895	94.25	400	23	23	1	400	1	1131	0	765	Pbre004909.1
PbreOR22	gnl|BL_ORD_ID|10738	25.417	240	179	3	159	395	298	1017	2.42E-21	93	Pbre010746.1
PbreOR23	gnl|BL_ORD_ID|1370	69.162	334	103	24	45	372	1	948	1.50E-147	421	Pbre001396.1
PbreOR24	gnl|BL_ORD_ID|1370	77.545	334	75	24	45	372	1	948	4.88E-178	499	Pbre001396.1
PbreOR25	gnl|BL_ORD_ID|20370	100	98	0	0	46	143	1	294	2.94E-43	145	Pbre020451.1
PbreOR26	gnl|BL_ORD_ID|15745	41.096	219	129	16	154	360	1	645	2.76E-41	152	Pbre015741.1
PbreOR27	gnl|BL_ORD_ID|10471	36.364	330	210	8	55	381	133	1107	3.17E-60	197	Pbre010528.1
PbreOR28	gnl|BL_ORD_ID|10738	30.068	296	207	16	99	380	139	1020	2.42E-42	150	Pbre010746.1
PbreOR29	gnl|BL_ORD_ID|17624	94.444	72	4	0	1	72	1	216	1.15E-43	145	Pbre017633.1
PbreOR30	gnl|BL_ORD_ID|4895	32.653	245	165	5	137	378	397	1125	6.62E-39	141	Pbre004909.1
PbreOR31	gnl|BL_ORD_ID|15745	52.381	168	80	6	154	315	1	504	5.77E-50	175	Pbre015741.1

PbreOR32	gnl|BL_ORD_ID|9243	89.607	356	37	35	24	379	295	1257	0	640	Pbre009306.1
PbreOR33	gnl|BL_ORD_ID|14717	97.902	143	3	0	234	376	1	429	4.59E-100	291	Pbre014668.1
PbreOR34	gnl|BL_ORD_ID|10471	99.458	369	2	0	8	376	1	1107	0	670	Pbre010528.1
PbreOR35	gnl|BL_ORD_ID|9243	100	99	0	0	136	234	1	297	3.88E-63	206	Pbre009306.1
PbreOR36	gnl|BL_ORD_ID|12418	61.29	124	48	0	236	359	34	405	2.74E-50	175	Pbre012401.1
PbreOR37	gnl|BL_ORD_ID|15745	46.784	171	91	8	154	319	1	504	8.08E-46	163	Pbre015741.1
PbreOR38	gnl|BL_ORD_ID|12417	82.143	280	50	1	76	355	1417	2253	1.13E-148	442	Pbre012455.1
PbreOR39	gnl|BL_ORD_ID|14708	96.346	301	11	0	71	371	4	906	0	562	Pbre014685.1
PbreOR40	gnl|BL_ORD_ID|9241	98.606	287	4	0	70	356	655	1515	0	586	Pbre009340.1
PbreOR41	gnl|BL_ORD_ID|9449	59.567	277	112	34	76	352	2296	3024	1.19E-97	311	Pbre009388.1
PbreOR42	gnl|BL_ORD_ID|2523	35.316	269	174	45	1	249	115	846	3.44E-35	133	Pbre002513.1
PbreOR42	gnl|BL_ORD_ID|12416	19.649	285	229	34	39	321	130	888	8.03E-07	48	Pbre012411.1
PbreOR43	gnl|BL_ORD_ID|10471	39.437	355	215	8	19	366	46	1107	3.10E-89	272	Pbre010528.1

PbreOR44	gnl|BL_ORD_ID|14712	56.69	284	123	0	1	284	1	852	3.43E-102	302	Pbre014676.1
PbreOR45	gnl|BL_ORD_ID|9241	93.182	44	3	0	182	225	1	132	4.20E-18	84	Pbre009340.1
PbreOR46	gnl|BL_ORD_ID|2523	42.667	75	43	0	1	75	1045	1269	9.97E-15	69	Pbre002513.1
PbreOR47	gnl|BL_ORD_ID|17211	25.49	357	266	16	3	348	19	1074	6.69E-16	76	Pbre017215.1
PbreOR48	gnl|BL_ORD_ID|9242	100	99	0	0	154	252	1	297	7.56E-68	210	Pbre009214.1
PbreOR48	gnl|BL_ORD_ID|12416	25.828	151	112	10	152	301	469	894	4.07E-06	46	Pbre012411.1
PbreOR49	gnl|BL_ORD_ID|10738	31.692	325	222	14	20	331	49	1020	2.83E-54	180	Pbre010746.1
PbreOR50	gnl|BL_ORD_ID|14707	86.471	170	23	19	1	170	178	630	7.91E-99	297	Pbre014702.1
PbreOR51	gnl|BL_ORD_ID|14713	76.531	294	69	34	6	299	1558	2337	5.74E-144	422	Pbre014727.1
PbreOR52	gnl|BL_ORD_ID|2525	47.766	291	152	1	2	292	175	1044	3.62E-102	303	Pbre002613.1
PbreOR53	gnl|BL_ORD_ID|9241	42.308	104	60	1	178	281	1594	1902	4.93E-25	103	Pbre009340.1
PbreOR54	gnl|BL_ORD_ID|2528	99.647	283	1	0	1	283	1	849	0	580	Pbre002605.1
PbreOR55	gnl|BL_ORD_ID|6306	34.524	84	55	0	187	270	964	1215	5.62E-12	64	Pbre006273.1

PbreOR56	gnl|BL_ORD_ID|2524	96.296	270	10	0	1	270	1	810	7.97E-157	441	Pbre002587.1
PbreOR58	gnl|BL_ORD_ID|14713	59.813	214	86	1	1	214	58	696	2.15E-84	266	Pbre014727.1
PbreOR59	gnl|BL_ORD_ID|2527	98.995	199	2	0	48	246	1	597	3.91E-132	370	Pbre002574.1
PbreOR60	gnl|BL_ORD_ID|14707	94.595	111	6	0	120	230	787	1119	2.70E-84	224	Pbre014702.1
PbreOR61	gnl|BL_ORD_ID|17624	35.211	71	46	0	4	74	4	216	3.71E-11	56	Pbre017633.1
PbreOR62	gnl|BL_ORD_ID|19144	97.934	242	5	0	1	242	1	726	8.77E-176	481	Pbre019187.1
PbreOR63	gnl|BL_ORD_ID|9241	76.023	171	41	35	69	239	1501	1908	1.96E-82	257	Pbre009340.1
PbreOR64	gnl|BL_ORD_ID|14724	49.032	155	79	0	1	155	679	1143	2.24E-51	169	Pbre014728.1
PbreOR65	gnl|BL_ORD_ID|2525	60.119	168	67	0	2	169	715	1218	3.27E-66	206	Pbre002613.1
PbreGR1	gnl|BL_ORD_ID|5675	41.139	158	93	2	264	419	223	696	6.59E-39	139	Pbre005644.1
PbreGR2	gnl|BL_ORD_ID|15159	99.254	402	3	0	446	847	1	1206	0	739	Pbre015110.1
PbreGR4	gnl|BL_ORD_ID|9593	100	227	0	0	94	320	304	984	2.48E-156	440	Pbre009583.1
PbreGR5	gnl|BL_ORD_ID|20522	62.245	98	37	0	131	228	40	333	1.01E-40	136	Pbre020487.1

PbreGR8	gnl|BL_ORD_ID|5675	100	134	0	0	1	134	310	711	2.45E-94	270	Pbre005644.1
PbreGR8	gnl|BL_ORD_ID|2115	99.074	108	1	0	1	108	667	990	4.08E-73	220	Pbre002124.1
PbreGR9	gnl|BL_ORD_ID|15160	34.615	104	68	7	13	110	844	1152	2.26E-06	43	Pbre015172.1
PbreGR10	gnl|BL_ORD_ID|8425	100	100	0	0	1	100	73	372	4.85E-63	185	Pbre008520.1
PbreIR1	gnl|BL_ORD_ID|14841	96.057	634	25	24	263	896	1	1830	0	1201	Pbre014850.1
PbreIR2	gnl|BL_ORD_ID|162	86.006	343	48	1	304	645	1	1029	0	614	Pbre000161.1
PbreIR3	gnl|BL_ORD_ID|18492	100	389	0	0	271	659	1	1167	0	762	Pbre018514.1
PbreIR4	gnl|BL_ORD_ID|19862	56.18	445	195	2	169	611	4	1338	0	519	Pbre019844.1
PbreIR5	gnl|BL_ORD_ID|19862	100	379	0	0	167	545	1	1137	0	765	Pbre019844.1
PbreIR6	gnl|BL_ORD_ID|18492	20.482	415	330	81	76	474	79	1128	1.80E-15	76	Pbre018514.1
PbreIR7	gnl|BL_ORD_ID|2243	99.558	453	2	0	1	453	1195	2553	0	907	Pbre002218.1
PbreIR8	gnl|BL_ORD_ID|17066	99.517	207	1	0	228	434	1	621	2.23E-154	434	Pbre017044.1
PbreIR9	gnl|BL_ORD_ID|18304	98.095	420	8	0	1	420	346	1605	0	781	Pbre018220.1

PbreIR9	gnl|BL_ORD_ID|14182	24	250	190	10	154	396	364	1104	4.52E-13	68	Pbre014213.1
PbreIR10	gnl|BL_ORD_ID|15627	38.739	333	204	3	18	347	4	1002	1.91E-88	269	Pbre015619.1
PbreIR11	gnl|BL_ORD_ID|19862	29.167	264	187	26	119	357	346	1134	1.34E-10	61	Pbre019844.1
PbreIR12	gnl|BL_ORD_ID|13495	100	280	0	0	15	294	1459	2298	0	585	Pbre013416.1
PbreIR13	gnl|BL_ORD_ID|2243	99.313	291	2	0	1	291	1	873	0	583	Pbre002218.1
PbreIR15	gnl|BL_ORD_ID|5082	63.426	216	79	0	1	216	631	1278	5.69E-99	298	Pbre005091.1
PbreIR16	gnl|BL_ORD_ID|18304	46.875	192	102	4	7	195	952	1524	1.68E-49	167	Pbre018220.1
PbreIR17	gnl|BL_ORD_ID|5082	52.121	165	79	2	2	166	1243	1731	3.66E-42	147	Pbre005091.1
PbreIR18	gnl|BL_ORD_ID|14840	36.538	104	66	4	9	110	328	633	8.59E-11	56	Pbre014805.1
PbreIR19	gnl|BL_ORD_ID|3919	100	143	0	0	1	143	817	1245	5.52E-99	293	Pbre003908.1
PbreIR20	gnl|BL_ORD_ID|15627	96	125	5	0	1	125	556	930	1.25E-84	249	Pbre015619.1
PbreSNMP1	gnl|BL_ORD_ID|11532	99.609	256	1	0	316	571	1	768	1.07E-171	485	Pbre011591.1
PbreSNMP2	gnl|BL_ORD_ID|7721	29.039	458	325	23	39	479	64	1419	2.58E-60	205	Pbre007727.1

PbreSNMP3	gnl|BL_ORD_ID|5560	98.23	113	2	0	1	113	490	828	2.43E-76	231	Pbre005577.1
PbreSNMP4	gnl|BL_ORD_ID|5557	100	26	0	0	26	51	653	730	4.11E-19	54	Pbre005589.1

Table S4. The BLASTx match of *P. brevitarsis* candidate CSP and OBP genes

Name	ORF (aa)	Status	Signal peptide	Best Blast Match				
				Blastx besthit	Species	Evalue	Identify (%)	Accession
PbreOBP1	156	Complete ORF	1-17	odorant binding protein 26	*Holotrichia oblita*	2.00e35	46	AZL87168.1
PbreOBP2	147	Complete ORF	1-16	odorant binding protein 5	*Anomala corpulenta*	2.00e71	80	AKC58526.1
PbreOBP3	142	Complete ORF	1-20	OBP3	*Holotrichia parallela*	3.00e62	71	AKI84361.1
PbreOBP4	136	Complete ORF	1-15	OBP12	*Holotrichia parallela*	2.00e37	46	AKI84370.1
PbreOBP5	136	Complete ORF	1-17	odorant binding protein 7like protein	*Anomala corpulenta*	2.00e79	82	AIZ03625.1
PbreOBP6	135	Complete ORF	1-16	OBP12	*Holotrichia parallela*	3.00e44	51	AKI84370.1
PbreOBP7	134	Complete ORF	1-25	odorant binding protein 3	*Hylamorpha elegans*	8.00e48	59	AMR98354.1
PbreOBP8	133	Complete ORF	1-22	PBP/GOBP family	*Popillia japonica*	1.00e52	69	KAK9731271.1
PbreOBP9	132	Complete ORF	1-18	odorant binding protein 9	*Anomala corpulenta*	3.00e45	57	AKC58530.1
PbreOBP10	130	Complete ORF	1-16	odorant binding protein 7	*Holotrichia oblita*	4.00e25	40	AZK90211.1
PbreOBP11	128	3' missing	1-18	odorant binding protein 27	*Holotrichia parallela*	4.00e73	85	ALP75940.1
PbreOBP12	125	Complete ORF	1-19	PBP/GOBP family	*Popillia japonica*	3.00e15	32	KAK9687289.1
PbreOBP13	112	3' missing	1-19	odorant binding protein 6	*Anomala corpulenta*	5.00e26	53	AKC58527.1
PbreCSP1	241	Complete ORF	1-20	chemosensory proteinrelated	*Holotrichia oblita*	1.00e65	78	KAI4469155.1
PbreCSP2	136	Complete ORF	1-20	chemosensory proteinlike protein	*Anomala corpulenta*	5.00e25	59	AIZ03628.1
PbreCSP3	131	Complete ORF	1-20	chemosensory proteinrelated	*Holotrichia oblita*	1.00e52	91	KAI4457662.1
PbreCSP4	107	Complete ORF	1-17	chemosensory protein 14like protein	*Anomala corpulenta*	7.00e53	70	AIZ03626.1

Table S5. The BLASTx match of *P. brevitarsis* candidate OR, IR, GR and SNMP genes

Name	ORF (aa)	Status	TMD	Best Blast Match				
				Blastx besthit	Species	Evalue	Identify (%)	Accession
PbreORCO	476	Complete ORF	7	olfactory coreceptor	*Protaetia brevitarsis*	0.00e+00	100	QRF71048.1
PbreOR1	455	Complete ORF	6	odorant receptor	*Protaetia brevitarsis*	0.00e+00	89	QBB72944.1
PbreOR2	430	Complete ORF	7	odorant receptor	*Protaetia brevitarsis*	0.00e+00	98	QBB72933.1
PbreOR3	422	Complete ORF	7	odorant receptor	*Protaetia brevitarsis*	0.00e+00	98	QBB72937.1
PbreOR4	422	Complete ORF	5	odorant receptor	*Protaetia brevitarsis*	0.00e+00	96	QBB72939.1
PbreOR5	422	Complete ORF	3	odorant receptor	*Protaetia brevitarsis*	0.00e+00	99	QBB72936.1
PbreOR6	421	Complete ORF	2	odorant receptor	*Protaetia brevitarsis*	0.00e+00	99	QBB72938.1
PbreOR7	419	Complete ORF	6	odorant receptor	*Protaetia brevitarsis*	0.00e+00	100	QBB72940.1
PbreOR8	416	Complete ORF	6	odorant receptor	*Protaetia brevitarsis*	0.00e+00	100	QBB72943.1
PbreOR9	414	Complete ORF	6	odorant receptor	*Protaetia brevitarsis*	0.00e+00	99	QBB72946.1
PbreOR10	414	Complete ORF	8	odorant receptor	*Protaetia brevitarsis*	0.00e+00	66	QBB72944.1
PbreOR11	412	Complete ORF	6	odorant receptor	*Protaetia brevitarsis*	0.00e+00	99	QBB72988.1
PbreOR12	411	Complete ORF	7	odorant receptor 14	*Anomala corpulenta*	0.00e+00	74	AKC58549.1
PbreOR13	410	Complete ORF	7	odorant receptor	*Protaetia brevitarsis*	0.00e+00	100	QBB72948.1
PbreOR14	410	Complete ORF	5	odorant receptor	*Holotrichia oblita*	1.00e169	64	KAI4456259.1
PbreOR15	410	Complete ORF	4	odorant receptor	*Protaetia brevitarsis*	0.00e+00	100	QBB72949.1
PbreOR16	409	Complete ORF	4	odorant receptor	*Protaetia brevitarsis*	0.00e+00	99	QBB72951.1
PbreOR17	409	Complete ORF	5	odorant receptor	*Protaetia brevitarsis*	0.00e+00	99	QBB72979.1
PbreOR18	408	Complete ORF	4	odorant receptor	*Protaetia brevitarsis*	0.00e+00	100	QBB72952.1
PbreOR19	404	Complete ORF	6	odorant receptor	*Protaetia brevitarsis*	0.00e+00	100	QBB72956.1

PbreOR20	402	Complete ORF	7	odorant receptor	*Protaetia brevitarsis*	0.00e+00	98	QBB72958.1
PbreOR21	400	Complete ORF	3	odorant receptor	*Protaetia brevitarsis*	0.00e+00	43	QBB72959.1
PbreOR22	398	Complete ORF	6	odorant receptor	*Protaetia brevitarsis*	0.00e+00	100	QBB72989.1
PbreOR23	390	Complete ORF	6	odorant receptor	*Protaetia brevitarsis*	0.00e+00	94	QBB72934.1
PbreOR24	390	Complete ORF	7	odorant receptor	*Protaetia brevitarsis*	0.00e+00	100	QBB72985.1
PbreOR25	382	Complete ORF	6	odorant receptor	*Protaetia brevitarsis*	0.00e+00	98	QBB73000.1
PbreOR26	382	Complete ORF	7	odorant receptor	*Protaetia brevitarsis*	0.00e+00	99	QBB72968.1
PbreOR27	382	Complete ORF	6	odorant receptor	*Protaetia brevitarsis*	0.00e+00	99	QBB72970.1
PbreOR28	382	Complete ORF	5	odorant receptor	*Protaetia brevitarsis*	0.00e+00	92	QBB72941.1
PbreOR29	381	Complete ORF	7	odorant receptor 9	*Anomala corpulenta*	2.00e169	63	AKC58544.1
PbreOR30	381	Complete ORF	6	odorant receptor	*Protaetia brevitarsis*	0.00e+00	99	QBB72973.1
PbreOR31	380	3' missing	7	7tm Odorant receptor	*Popillia japonica*	2.00e104	47	KAK9727595.1
PbreOR32	379	Complete ORF	5	odorant receptor	*Protaetia brevitarsis*	0.00e+00	100	QBB72997.1
PbreOR33	376	Complete ORF	6	odorant receptor	*Protaetia brevitarsis*	0.00e+00	98	QBB72976.1
PbreOR34	376	Complete ORF	5	odorant receptor	*Protaetia brevitarsis*	0.00e+00	99	QBB72977.1
PbreOR35	376	Complete ORF	5	odorant receptor	*Protaetia brevitarsis*	0.00e+00	100	QBB72959.1
PbreOR36	373	Complete ORF	5	odorant receptor	*Protaetia brevitarsis*	0.00e+00	99	QBB72993.1
PbreOR37	373	Complete ORF	6	odorant receptor	*Protaetia brevitarsis*	2.00e110	49	QBB72958.1
PbreOR38	373	Complete ORF	7	odorant receptor	*Protaetia brevitarsis*	0.00e+00	80	QBB72980.1
PbreOR39	371	Complete ORF	6	odorant receptor	*Protaetia brevitarsis*	2.00e143	69	QBB72996.1
PbreOR40	371	Complete ORF	6	odorant receptor	*Protaetia brevitarsis*	0.00e+00	99	QBB72972.1
PbreOR41	370	Complete ORF	6	odorant receptor	*Protaetia brevitarsis*	0.00e+00	100	QBB72982.1

PbreOR42	369	Complete ORF	7	odorant receptor	*Protaetia brevitarsis*	2.00e136	60	QBB72937.1
PbreOR43	366	Complete ORF	5	odorant receptor 19	*Holotrichia parallela*	1.00e87	41	AVH87260.1
PbreOR44	365	Complete ORF	3	odorant receptor	*Protaetia brevitarsis*	0.00e+00	99	QBB72981.1
PbreOR45	360	Complete ORF	7	odorant receptor	*Protaetia brevitarsis*	0.00e+00	100	QBB72978.1
PbreOR46	350	Complete ORF	4	odorant receptor	*Protaetia brevitarsis*	0.00e+00	99	QBB72947.1
PbreOR47	349	3' missing	6	odorant receptor	*Protaetia brevitarsis*	0.00e+00	100	QBB72961.1
PbreOR48	334	Complete ORF	4	odorant receptor	*Protaetia brevitarsis*	2.00e177	100	QBB72992.1
PbreOR49	334	Complete ORF	6	odorant receptor	*Protaetia brevitarsis*	0.00e+00	100	QBB72964.1
PbreOR50	306	Complete ORF	2	odorant receptor	*Protaetia brevitarsis*	0.00e+00	84	QBB72983.1
PbreOR51	299	Complete ORF	5	odorant receptor	*Protaetia brevitarsis*	3.00e168	97	QBB73001.1
PbreOR52	292	3' missing	4	odorant receptor	*Protaetia brevitarsis*	0.00e+00	99	QBB72975.1
PbreOR53	284	Complete ORF	5	odorant receptor	*Protaetia brevitarsis*	8.00e91	97	QBB72971.1
PbreOR54	283	3' missing	4	odorant receptor	*Protaetia brevitarsis*	0.00e+00	100	QBB72967.1
PbreOR55	278	Complete ORF	3	odorant receptor	*Protaetia brevitarsis*	2.00e116	99	QBB72994.1
PbreOR56	270	Complete ORF	4	odorant receptor	*Protaetia brevitarsis*	0.00e+00	92	QBB72935.1
PbreOR57	262	3' missing	4	odorant receptor	*Protaetia brevitarsis*	0.00e+00	100	QBB72955.1
PbreOR58	254	3' missing	5	odorant receptor	*Protaetia brevitarsis*	0.00e+00	100	QBB72984.1
PbreOR59	247	3' missing	3	odorant receptor	*Protaetia brevitarsis*	0.00e+00	98	QBB72953.1
PbreOR60	245	Complete ORF	3	odorant receptor	*Protaetia brevitarsis*	5.00e176	100	QBB72983.1
PbreOR61	245	Complete ORF	3	odorant receptor 9	*Anomala corpulenta*	1.00e40	36	AKC58544.1
PbreOR62	242	Complete ORF	1	hypothetical protein QE152_g22519	*Popillia japonica*	2.00e27	34	KAK9719704.1
PbreOR63	212	3' missing	3	odorant receptor	*Protaetia brevitarsis*	1.00e37	37	QBB72976.1
PbreOR64	170	3' missing	2	odorant receptor	*Protaetia brevitarsis*	3.00e65	60	QBB72966.1
PbreOR65	166	3' missing	0	odorant receptor	*Holotrichia oblita*	2.00e59	64	KAI4456945.1
PbreIR1	896	Complete ORF	3	ionotropic receptor	*Protaetia brevitarsis*	0.00e+00	100	QBB73017.1
PbreIR2	747	Complete ORF	3	glutamate receptor ionotropic, kainate	*Tenebrio molitor*	0.00e+00	85	XP_068900418.1

				2like isoform X2				
PbreIR3	722	Complete ORF	4	ionotropic receptor	*Protaetia brevitarsis*	0.00e+00	100	QBB73020.1
PbreIR4	625	Complete ORF	3	chemosensory ionotropic receptor 75q	*Anomala corpulenta*	0.00e+00	63	AKC58589.1
PbreIR5	545	Complete ORF	2	chemosensory ionotropic receptor 75q	*Anomala corpulenta*	0.00e+00	50	AKC58589.1
PbreIR6	473	Complete ORF	4	Ligated ion channel Lglutamate and glycinebinding site	*Popillia japonica*	0.00e+00	68	KAK9711344.1
PbreIR7	453	Complete ORF	4	ionotropic receptor	Protaetia brevitarsis	0.00e+00	100	QBB73018.1
PbreIR8	434	Complete ORF	4	ionotropic receptor	Protaetia brevitarsis	0.00e+00	100	QBB73022.1
PbreIR9	420	Complete ORF	3	chemosensory ionotropic receptor 75x	*Anomala corpulenta*	5.00e156	53	AKC58588.1
PbreIR10	378	Complete ORF	3	chemosensory ionotropic receptor 41a	*Anomala corpulenta*	0.00e+00	64	AKC58587.1
PbreIR11	358	Complete ORF	3	ionotropic receptor	*Protaetia brevitarsis*	0.00e+00	100	QBB73024.1
PbreIR12	294	Complete ORF	1	ionotropic receptor 1	*Holotrichia parallela*	2.00e162	77	AVH87289.1
PbreIR13	291	3'	0	ionotropic receptor	*Protaetia brevitarsis*	0.00e+00	100	QBB73018.1
PbreIR14	270	Complete ORF	2	glutamate receptor ionotropic, kainate 2 isoform X1	*Periplaneta americana*	2.00e131	96	XP_069680346.1
PbreIR15	216	3'	2	ionotropic glutamate receptor	*Holotrichia oblita*	7.00e122	81	KAI4458353.1
PbreIR16	200	Complete ORF	1	chemosensory ionotropic receptor 75x	*Anomala corpulenta*	2.00e60	52	AKC58588.1
PbreIR17	166	Complete ORF	1	glutamate receptor ionotropic, kainate 2like	*Onthophagus taurus*	2.00e67	67	XP_022911458.1
PbreIR18	146	3'	0	ionotropic receptor	*Protaetia brevitarsis*	6.00e98	99	QBB73023.1
PbreIR19	143	3'	0	ionotropic receptor	*Protaetia brevitarsis*	6.00e93	100	QBB73021.1
PbreIR20	126	Complete ORF	0	ionotropic receptor 3	*Holotrichia parallela*	1.00e33	50	AVH87291.1
PbreGR1	441	Complete ORF	6	gustatory receptor	*Protaetia brevitarsis*	0.00e+00	99	QBB73006.1
PbreGR2	439	Complete ORF	4	gustatory receptor	*Protaetia brevitarsis*	0.00e+00	100	QBB73005.1
PbreGR3	402	Complete ORF	7	gustatory receptor	*Protaetia brevitarsis*	0.00e+00	100	QBB73007.1

PbreGR4	320	3' missing	4	gustatory receptor	*Protaetia brevitarsis*	1.00e136	100	QBB73010.1
PbreGR5	236	Complete ORF	3	gustatory receptor	*Protaetia brevitarsis*	1.00e124	100	QBB73009.1
PbreGR6	236	3' missing	4	gustatory receptor	*Protaetia brevitarsis*	2.00e164	100	QBB73008.1
PbreGR7	137	Complete ORF	2	gustatory receptor	*Protaetia brevitarsis*	3.00e92	100	QBB73008.1
PbreGR8	134	Complete ORF	0	gustatory receptor 2	Holotrichia oblita	6.00e79	92	UNE74349.1
PbreGR9	123	Complete ORF	2	putative gustatory receptor 2a	Lucilia cuprina	2.00e04	55	KAI8116626.1
PbreGR10	100	Complete ORF	0	gustatory receptor 8	*Holotrichia parallela*	7e18	66	AVH87323.1
PbreSNMP 1	571	Complete ORF	3	Snmp1	*Trypoxylus dichotomus*	0.0	87	GJQ65243.1
PbreSNMP 2	547	Complete ORF	2	sensory neuron membrane protein 2	*Holotrichia parallela*	0.0	55	AVM18970.1
PbreSNMP 3	113	3' missing	0	Snmp2	*Trypoxylus dichotomus*	2e29	50	GJQ79424.1
PbreSNMP 4	104	3' missing	0	Snmp2	*Trypoxylus dichotomus*	3e12	53	GJQ79425.1

File S1. The amino acid sequences of *P. brevitarsis* putative chemosensory receptor genes.

OBP

>PbreOBP1
MLKIVVCVLLFAIAVQGKSALRKSLNEQMAQMSNECVQQTGMDRQVLQKVFVEEQMPEK
SAIYLTFLECLYKKQNYVDDDGLISYPAIENFLKQFIDSGDLRTVMAPCEKLQEGTSIGEKAF
NAGHCILKQLHVLEARKNTESSESAESSEEANKV
>PbreOBP2
MKVILFLVYAVALIQAKRKLTEDEIIEANKNCLKSTGMDGAVIKNIISYDTFPKPSNNYFKYL
ECMYINQGYLDSDGLISYQTIEDFILDFYDLDTVKLAIEPCIVIQDGKNGGERAYNAAKCLIR
NLEALEKRYEKQNKGVPEKSK
>PbreOBP3
MVLNLRICFLIVVGLGSVLSLNEAQMKAGVKLVRNTCRSKTKITDEQIEKMHQGVWDDDD
EVTKCYVHCCLGMMKMQTKSGGFDRALADKQIPQLPDSYKVTLVKSFDNCINAGEGLTK
KCDLAYAFFKCVYFDDPEHYILP
>PbreOBP4
MKCLIVLLTVITISATMTTEEKNELKTLAKDCQKESGVEKELLTKMKGGDLVDDPKFKSFLY
CLYKKINFYRPDGTVDMDVVRSRFPKDMTEDQKNAIINDCLSLKGTDAKDTAYLHFKCYR
NKTSKTNDVDVLTD
>PbreOBP5
MKTIAFVVLLFASSILCQESLQERQQRTQKYREECVQESKVDPAVIDKADEGDFADTRELKC
FARCFYTKAGFLTEQGELLLDVVKAKIPAEHNREKALAIIELCKDLKGSDPCETAYAIHKCYF
QNAHAANLHKN
>PbreOBP6
MKCLIVLFAVIAISVAMTPEEKNEIRTLAKECRTESGVEKEVLTKLKDGDVVDDPKLKSFLYC
LYKKTKFYRADGTVDTDRVRSKLPQDLTDAQKDAIINECLSIKGTDAKDTAYLHYKCYRNK
SSKNNVDLLTP
>PbreOBP7
MENWNGIFFTLVFVNLILLHGLVSGGDSSSDLLHKNICIRETNVNPALVEKAENGDFPGNR
ELQCYYRCYYIESGFVNNLGEIQTDVIKSKIPQKTDKEIAQHAIDKCKRVKGMDSCETAYQI
QKCLYNNNLKL
>PbreOBP8
MKKVIFILPLIFTFARTFSVESQNLAIAKCIQKTDIPESTATRMGITLEIPDTNIAKKFLICLNKK
MGFQDEEGQILFDNMKNSLTGLTEAEVNSTINTCKQVKGTNSIENSYLATKCIFRKIKSIIKL
KKRK
>PbreOBP9
MKFFIVIGVLCAIVYVKALTDEQKLLAKQNHDKCVAESGVDIELVEKGRKGEFVVNPKLRE
FLLCFLKASELVDDNANPRPDKIKEKLGQDLTEAEINEVITKCQSNANTPQEKAAAFYVCY
WATSKHHVEF
>PbreOBP10
MRQIIFVLFFMSAAFAQDLLTDVDDETKECLKELNMKSDQVRNLDSLEEISKMTKDEKCLL
RCVMKKVGIIDEEGNLVEEELMDDEILESMDIDVSKCTLQTAITDPCEQNYLLTVCVVEVMT
EAAAREN
>PbreOBP11

MKYNIVLLLFAIYTYSEGLLSQTEQIEAVQLCANRTGLAPKSIGKLTRKLSVPKNDPAVKTFL
VCVYKYVGLQDQQGNMNFEKIREQLKNDFFKYDTDSVVDTCSTVRGQTHEDNAYRATQC
ILKQIR
>PbreOBP12
MKCLSWILFIAVYLTPTNAFFEDFRPYAEQCLTELDMSEDEVTLFKENVMRDMEVSHNGKC
LASCMGTKSGFLQGDKINMDVVKTLYPAAANQNFTMCESVTGSDDCDKRFKVIHCCLGIL
KDVI
>PbreOBP13
MNPTFLVLFLFAHLLFVSADEYETERNKLRQMGIKALMECKTKTGAPDADLQAALKKELP
TSKKGFCMLECFFNKANVMKDGKLSVDGTLKTMESALSKNADVDRKVRRVLQ
CSP
>PbreCSP1
MVLVTVLLLVFGLEIATVIGADPPGGYYTTRYDHIDIENILNHKRLVLYYAACLLDRGPCTPQ
GTEFKNILPEAIKTNCLRCTEKQRSVTVHSIRRLRKEYPDIWLQLEKRWDPTGENVKRLLAS
ANKPIPVPTETALIADRFGGVEVTIQTSSGEQGRIAPIPTETTTIQAANTTTISSTVTTTTPTITTI
FKGTTKTVTSRPFNSVGPNFIVLNPKALIDKVILTADVVLKTVTGVFEG
>PbreCSP2
MLQLRTVLCFMAVIFLGVDAQQYNTKYDDFDIDNLIKNDRLLQKYVECLLDLVACTVEGL
ELKRNMPDAIQTNCTKCSEKQKIGSEKLISFLIDNKPELWEPLEKKYDATGEYTQRFLDLRK
TTTAEADQSTFKSN
>PbreCSP3
MKVTLLICVFLFDSFRTSMTQNSPGVFLSKYKVDVDNVISSKRLLINYSNCLLEKGPCTVEAR
ELKKIIPNALATQCAECSDDEKKAVGKIFSHLLQNHRDLWNQLLDKFDPDGTFRKQYELDE
EDYDDEE
>PbreCSP4
MRFTQIIFTCIIAVAIAKPQERPQISEEALDRALKDHRYLMRQLKCAIGEAPCDPVGRRLKSL
APLVLRGACPQCSPSETKQIQKVLGYVQKNYPREWNKILQQYAG

OR
>PbreORco
MMQFKPQGLVADLMPNIKLIKFSGHFMLNYYADNSGAVHTLRLGFCFAHLFLLLLQYGFTF
GNLVKESDDVNDLAANTITVLFFTHCITKFIYFAVRQKLFYRTLGIWNQSNSHPLFLESNNR
YHQLALTKMRRLLIVVMVGTMTSWIAWTTITFFGDSVHTRKDPNNENETITEEVPRLLVRS
WYPWDAMSGVPYYISLVYQIYYVGFSMLHSNLLDSLFCSWLIFACEQLQHLKEIMKPLMEL
SATLDTYVPKSADLFRAPSAASRDNLIDNDYNQRNEEATMKGFYTTSQEMGVTYRSGNIQ
DFSGGIGPNGLTKKQELMVRSAIKYWVERHKHVVRLVTAIGDAYGIALLLHMLTSTITLTLL
AYQATKIDGVNKYALTVLGYLIYALAQVFHFCIFGNRLIEESSSVMEAAYSCHWYDGSEEAK
TFVQIVCQQCQKAMSISGAKFFTISLDLFASVLGATVTYFMVLVQLK
>PbreOR1
MAGGIRSWIRAYKQKELPKNYFRAQVKILRFLGVELMRDETIKYRIYSCIMLFIVAIQGTFSE
AVEFYAQWGDLNAIVNVTSYLFTHSIGIIKVMMLFYNRKKFGKMIKTLHNKPFAPEVNRGG
DIEEKYLRQIVRTTERQLMFFFVLTYVTLFTAAVTFIKSRVFDPKEEWQYPYVPITIIKTTDSPLF
EMVGVYEVIWVSLYAGLLITTDILLVTILAHLTIQFMILNNAFKTIRTRSKRMNQNADGTDE
NEGVILSKLLGEYIEHHLRVLELAEEMEELCNMVYLLIFTSSVILLCFLLFQMSLVSRTFSNYP

NIIHYVRSPTLLITPTSYIPYIFASINRKSFQYPIGSVEFIHNISYYGIIAYQIGMYCFWGNEVTLQ
AEEVAVAVAEADWVGAPRSVMKAMVLIIARSQKPLYMTAGKFVNLSIDTFTRIVKGSFSYYV
VLQQTQEDTN
>PbreOR2
MSSQRLLKDYFKVQILFFKMIGVNLENSDNSFKSRVYFVYSQVILILTVYLFFITECVDLIGKW
GDIDNMTFNLCYLLSQTAGICKIVAIMLQREKVKKMLKILESDYFLPNYDRGGITEYRIIKEA
VRQINVQTYFLNTIVCLIVANRGFFAGFDKGYFEPTDDFSGNGTVKHYQLLPYTTWFPFDT
NITPFYEIAFIYQIAGALMGGLVIGTSDSFICGLMVHVKAQLRILKNNLNNHVKKAIREKQI
NTGINKQVPNITLYDTKKLLPEVQALIKHYLGECIIHHQKIIHLAERVESDFSYIMLIQFLTSL
GLLCLSMFQISNNNVKSTGFLSMACFSFMMFYQLFMFCWNGNGVLELSLDIVFAAYDSEW
FLCDLEIQKAIISIIQRAQRPLQISAGKFAFLTLETYMNILRASGSYYMVLQNVNQ
>PbreOR3
MPFLRRLHPFLKRFRFLQESAHFLADASKYSLVLAQIYLYAANMWPINFTSVRKTLFFTVIGI
AILHLLSILVYCYSYVKNVDTLAEVFQQVDLGIQVLYKSSVVFRKSNRLSNLIHIVWYEFWPS
NLFGGELEEKIADDTKTVIVPILLEIIVAGIYSAICLFLPLFAKSTLLLHEAWYPPGWDRNPMF
KYAHIFKSFVAVYTDLGIICAYDLLYVILCTNCSAQFRLLCSAMELIGSGMEKDTVDDIQGDD
NDIRNPSSHGTREEYEMELLSICVKHHQKLIQFGDELNMIFGSGHFLQLHTTLMGICLTSYLI
ASADNLIYIIFNIIYYVAHFWQLYVFCAASNQVSDSSVCVLDAAYSSRWYTKNYSDITKCLIM
IMIRSKSPITMNAFGLIVVNYVLFLTVMRFSFTLYTFLRTLAA
>PbreOR4
MIFKNRRNPVLKLGLGLLKADEDILEDDVKYTMVISKIILSTVHLWPENNNVYTKLSFYMIF
FVYASIELSLLVHIFVAVKDVVALTDTMLMFSVGVQTLTKAAILYLKSEDLNKMIQSIRKEFW
PSNSFGEDIKIQIKSETRKLLYLCLTSYIGGLCFLLQFILRPVVEATRVLPYNSWYPFNWSKTPI
YEILYILQGYMAVIVNLNIVFGTDCLYYFICANCTAQFRLLCKAMKEIGRVRENVYSHILLKT
VGVTYSVISNEKSENERRLLVLCIQHHQKLLDISFRLNQLFSYANLCQLAASVAGICAACYLV
TSDSKASASSSAPYFIGHIAQLLLYCAVSNELGHWSQQLSIEAFKSGWYLQKCTDIRQCLSIL
MMRSQEGISMTALGLFELSYASFLTVVRFSFSLYTFLDKFKET
>PbreOR5
MMVFRKNVQRMLNRFQLFQDKVHFLNDEAKYAMVLGRLLVSIANLSPMDYNLARMLFL
LFTVAVSIIHEITIAVCFASYVRDVDTLTDVLPALSLGIQIVSKGTVLLIKTKQLATLTEVVWND
FWPANIMGHAVEYKISKLSKRILIAFYTELIVTIVYSISTVILPLRRTPKVLFQQASYPLDLSKPL
GYSIAFCIQSYMTLYAESATICGYDLMYQAICINCAAQFRLLCVALEYIGTGKEKEILEEIQNA
VSDKTKRISIKDQDANKELLGICVKHHQTLINVGHELNKIFGTGHFLQLCATLAGICTISYHI
SQTNDYNTIFVGLNYYFGHLCQLFMFCSASHELSFWSTCPSMSAYNSKWYNKGCDKIKKSL
QILMIRSQRQMTMNSFGLFELNYVSFLAVMRFSFTLYTFLKTVAD
>PbreOR6
MKSLRHRLLSFLARFEFFNSRMDMLKDKGKCVIFAGELPLKLAKLHPADRGASSIMYFLIIM
VLVTLHVGLMYACLLENSRVDAIANILGLSNGGMQTIFKATAIFYQRKKFTNILNTMRYSF
WPADAVDSATEEQIWTSSKKLLLLLSTQYVVPALYLIYIMIGPILKGSRYLPCAYCLLPNQTDE
SPIFEMVYAFQVITTVFLPLHIIGGNDNLYVTSCSICSAQFRLLRAAIRVVGSGHEGELIERLLR
LPGVETPPSRGRSDKAKILLVICIKHHQNLLRFIDTLNRVFENGIFGQFVFSLAGIWANCYTL
TIQTTIKGSVTTLAIYGSCLLQLLLFCAFSDDLSHLSADLANGAYESMWYQNGDPDVGRCL
ALVILRSQKAVCMNAYGLFELNHVSFMAVIRFSFSLYTFMSQVAT
>PbreOR7

MFFLKKLRYFLNRFDLVQDNVHFLEDASKYVMVLSRLLLHFVNLWPMNFTKIRKVFFYAT
MGIAMGHHFTIFSYTCRNVRSIDNLTEILPAMSLMIELLSKTFVLYGKRDQLKDIIKIVWNDF
WPSNTFGKELEEKISTNTKDILIPIILQMAVGSIYSTICIFLPFVATPSFVYHAWYPPGWDRSLF
QTILNILRSYLMTYVINSMVCAYDLLYTALCINCISQYLQLSYALKFIGTRKELEVILKIQGKD
NKNPSIIQNDEGRVMKLLIICAKHHQMLIRFGNELNTIFGSGHFLQLVATLIGICTTSYNISQK
TGHMVVMLSCGYYLAYFWQLYVYCASSNSLSVWSTYVSDAAFDSEWHASNTRDIRKCLSII
MIRSQQPVIMNAFGLFQLNYESLLTVMRFSFSLYTFLRKIAA
>PbreOR8
MESYTPNFFRVNAEFLTYSGLWSPPYKNGIKYYLYKLYKTVVLFSVLFYNPCVVILGVIEHIH
DFVIVIEALNVGLTILLTGVKSAFWLSNGERIKEIMHRLETNVLHYEETDGFDSVRMIREAQ
KSGLWYALLLWRAALLTLAFAYIPVAMLSLWYCFNDLPITGVKAFENLPYYMYVPFKRDTA
FKYFLACVLQCSPCFICATVFVGIDSLFMSMMNLIGTHMLIVQGAFRTSRKRCLKKLKGRAL
TEDGLYNSEELESYMMDEMKSCIEHLQMLFRSCSDIEDAFQYMSFVQALCTMYILCSTLLLIS
TVNVLTNEFGKDLIYFLGVTLQLGLYCWFGNQLTLKSEGVPLAMWESDWLETRKPYKFCM
LVTMMRFKKPVYINAGKFVPLILDTQIAVIKGSYSYYSVLKGMSK
>PbreOR9
MYDMQEKRKNGLHRLSFDFTKDMYQDKAKNCILPAKVLLESVCCWPDNTSSSRTITAWIL
FLNLMVAEIFHAAYVVVHASDISDAVSAGATVTTTFEALVRVHIMLSKKRVINEILSVIWKQF
WPLRVILKEEARKELKMKAMISVLLPCTFLVSSVLSNLLITGLPFIKEHQLVLKSIFPFDWNQS
YIYEALYVWQYVMDWYVLFMVNAFDFFFVSLVTICCIQFLIMQEVLTLILSSQSKEHRKIIFG
RAGENMSDHQMLWECLKQHKLLIGICDDIEQIFNKAALIQFAVSACANCAAFLIMKVDYA
QFFKMLFYAMAHLVQLFYYCYVGQQLSYESEALADAIYKCDWHLQYDRSFRKSLIMMIQR
SQRRVCLTAVGFVELDFGSFIRILRMTFSFYTLLNNLLMKNEQE
>PbreOR10
MGGGIRSWIRAYNQRGLPENYFRVEVYILRSLGIELMREETLNYSIYSRIVLFVIVYLCTFGETL
ELYAQWGDLNAIVTVTSYLSTHILGLIKVTLLFYNRKRFGRMIKILHNKPFAPDMNRGGKIE
EKYLRRIIKTTETQLIVYFSVVSVTICSGLVIFLKSRILNDKSDWRYPYAPITIFNTTYSPIFELVAL
YDVIWYLLYAAVIGATDVLLSTILAHLSIQFKLLNNAFKSIRTRSKKANELARGDPEDEGVVL
RKILGEYIQHHLRVYQLAEEMEELCNLMFLVVLLASVILLCFLLYQISLMPIGSVPFIQNFFYY
WIIVCQMGMFCYWGNEVTLQAVDVAKAVAEADWPGAPKDVSKAMVVVIARSQKSVHIT
AGKFVTLSAETFMSIIKGSFSYFMVLRQTQAREE
>PbreOR11
MGSIRSWIKAFNQKELPENYFRVEVFILRYYGIELMEKETLKSRFYAGIVFVLIYLCTFGEALE
LYVQWGDLDAIVNILCYSCTHILGLIKVTLLFYNRKKFGNMIKILHNKPFVPDINRGGEVEEI
YLRKIVKTTETQMIAYSTLLVTALWSGAVSFLNSRIFNEKSEWRYPFVPIMIIDTTNSPYFELAG
IYQTFWISFYGLLIVTADIVLTIILAHLSTQFKILNNAFKSIRMRSRKMNELAGGDSRNEGIILS
KILGEYIEHHLRVFELAAQMEELCHLMILAELSGSVLTLCFILYQVSSIPPNSFSFLLYFFYYWI
VVFQISLYCYWGNEVTLQAANVAKAVAEADWLEAPKSVRKAIILVTARSQKPLYMTAGKFV
NLSIDTLVRIIKGSFSYFMVLRQRGISEE
>PbreOR12
MNNFFDVNFTILKVAGIWVPGPNSSYRAKFLYFVYNSFWITYSCLIFCPSELAYFINTYTSLKD
LIKNVNMGMTHFLANIKVCMWFYYRKDIIGIIETLDIYGKRYESYGDFDNEKIVQKEKKFKD
KFSVIFLLFGMLTSISSCMACFYQTLKLKLQEDERVQLSLPYFSYIPFDYEYSKVKFLIAIWYQF
FPLFNYAYLIIGFDTLYIAILSYISAQLDIIYGAFETIRPRCMIRLKLDLPKNILKDPPMLMKEM

HKEINKITYHLQVILDICRRLEDIYSTIILAQVLISLIVLCTCIFLVSLLPMMSLNFAAEMIYLIAI
ECQLLIYCVFGNQVTVSSSNISKSLYTGDWYSTSTTFKRSMLITMTRMQKPIYFTIGKFTPLTLS
TFLTISRASYSLFAVLKNSDLGT
>PbreOR13
MEMGFFDLNVKILKLSGLWVPDKEDSNYMILVMYNSICVCYSMIYFTIAELVALKESASDLN
DLIANLNMTMSFILTLAKVFGWFYYRKNIVRIIKLLEAKENTFSENNVDNENIISEKKRFKNI
WTKSFFIVASLVPISAGILSLTDTVISGNKYINYQNDSTTIYLQKLPYHSWIPFNYASSKYSFSFA
VITQCLALLNCGYITVGLDMMFVALVSLVTAHFTVCSMAFEKSTDVRREISKQLKDSFVYQK
VLMRECDKRIKKCVKHLQVLIGVCQELETIYSPLVLLQVLISLVVLCTCLYLVSSLPIGMRLLG
NELAYLLAIEIQVATYSYVGNELTYSALEIPTAIYQSKWSSTSLDFKKIMLITMMRMQKPVHIS
VGKFSALTLNTFVMIAKTSYSIFAVLKGN
>PbreOR14
MVLPLLRYSNNEGPKNYFALQKGVLKILGISFSGDESRWYRIYSFVWLTSVIISFSVIELYELYIY
KDDMDVTINNLSYLGTDLLGIAKMSVLLYHRLNIGKMLDRIEEGVFSPNKLRGGDLEEELIK
KCILFSNRQTCLYYLSVGMVVFIGVVASLLKRHLEPEAENWEMPYTTFSWFDIHRSPNFEIV
CFYQFCWRGLYALIVSSIDSLIAGILAHISVQCKILQNGIKKSIPYSKGKSEEAQLLLDTVSIPR
QILARKMEEIVDYHLAIIDLASQFEELFNFLILTIFLATLFILCFVMYHASLFELLHPKAAQDFS
YVILVALQVCLYCYWGNEVRLESEKIADACWQLDFVGTEPQFQKSLSLVIRRSQKPIVLTAG
KFTTLSLETYVRIIRVSYSYYMVLRNYH
>PbreOR15
MEYKRLLQESGIPVHKLPKAFTSDLSKDAAKRCIIPGKWILQTILAWPDTDNLYVVAANWF
LFINMIVVEIFHATYTVVYRADLGNALLALATVTTTLEGLVRFQMIFFKKHLFNDILHKIWK
KFWPLSVLSSTKEVEILKRRCYTTLGLTIGCYGPAVICNVVLTLGPYLTGDGLILKSVYPFEWN
TTYAYEAVYTWQYFTQWYILILVNTFDSFAIPMMMICAVQFVVWQDIFRNIFTVGSKKQRLA
LFGKEVNDEEMISICLEQQNMLFEICQKLEEIFSFAILFQFGNSILALCSSCVILKVDQSKFFEM
FTFAVAHMFQLFNYCYVGSELAVQSENMAAAIYDCNWQDSDDLKFKKVLPFMLQRSQKV
RNLTAAGITPLDFISYVKVLRISFSFYTLLDTLFDSE
>PbreOR16
MSGKKTTLRKLSLEFTKDVYKDGVKKCILPGKILLQSVCCWPDDESLFMKVVGWFLFWNL
FVIEIFHAAYVFKNFNDIGDAVGAGATVTTTMEGLVRLHIMLTKRDVINITLVKIWKQFWSL
DVIEPIKRKQIQRKAQLSVMLTSIFLASSIISNSQITGVPYVRNRGMVLKSVFPFDWQKSYYYE
VVYVWQYYSDWFVLFMINAFDFFFVAMVTICAVQFVIMQEVFKSILGKDSRRQRVIMFGEK
GQKMTDRQMLFEALEQHKLLIGICNELEESFNRAILIQFFVSTSAICAASLVLKVDYSQFFKM
LMYAAAHLSQLFYYCFAGAELSYESGHLADAIYECNWHLSYDREFREAILMMIQRSQRVQ
CLTAVGITELNFESFLKIMRLSFSFYTLLNNLLMKHMDG
>PbreOR17
MLGEINNESEKLPSEFTKDMLKDDIKRCFIPIKIYLEALCCWPYNRSAFIKFCAWFIFSNMLIA
EIFHAAYVVTNIDDVGGAVGASATVITSFEALVRMYILLTKQDFIKEILLKISKQFWPLQAISN
VDTRNQLRRRVILSISLPFIYLTCATISTGGYVFMSLRSYQLVYISAFPFDWSKRFVYEIIYFWQ
YIINWYNIFMINVFDCFFVSIVAICTAQFVILQEALRVIFDEKSRRQRRIIFGMKGGYMTNREV
LLKCLEQHKMLLSICTDLETALQRATLIQLAVSVNASCSACLMMTIDYSQAAKMLSFGIAH
LIQLFYYCDVCQKLSTESEQLGDAIYESSWHLEYDRDIRRTLILMMQRSQRKVQMTAVGVM
ALNLSTFVRVLRLIFSLYTFMSAMLVRNDL
>PbreOR18

MGAYNTDLFYINDFFLKCAAVWPINHKTGIKHVSYKIYQFFVIAVTLVLFPTSLFFNVAQNV
DNLLTFMEILYPAVIGLLSAAKVYFSFMNCDRIKRVMYSLECNDFHYEKIENFDPCLIMKRA
KLRGIITTMTMWCLCQLTLFLTYGSPIVKSFWYYIKDMPIGNVTTFQTLPTRLHPLFQCDTAL
KYLIACLLQFILFSLYILVIVGFDGLFMNLLNIIGEHMVILQGAFRTIRNRCLLTISGIDLGVDE
LEERMMIEMKKCIRHLQMIFQCCADTEEIFKYICLIQSTATLLQFCDCLMLLSLTDFRSTEFR
MYASYSIAIVTELSLYCWSGNNLTSRAIDIPLGLWESDWLETRKPFKVCMLITMIRLQKPTIFT
AGNIVPLLLTTQVSILKAGYSYFTVLSGR
>PbreOR19
MHSNSQSSRLGDYFRPQKLILKICGISLRENESISYRLYSGFVMCMIVSLLFVEIHGIVVHIKDV
ENLAGFIAIVFQHVLTLFKLIILKRNRAIISETLQMLHEGCFAPNFERGGAIEEKLIKECNHDI
FVRSATYYIAGAITIGNAVIIALRTKLGQDDYRLWETFWLPFELFEIKSTTCYYIVYIYCSLAITY
FSTFVMVTDLLITSVIKHATTHFVILGNFIRNITKYKVEKNNDELYNDYPKLRNDRLRMRMR
AAIVYHQEIIKVTERFENLFNIFILCEFVGSIFVLCGTMYKSTLYSLGDQRTIRECVFLFTMTTQ
LFIYCYCGNELTLSSTSVSFACYEADFIDEDLWFQKNLLMIMMRSQKPVALTAGKFTVLSLPA
FVGILRVSYSSAMVLRKAN
>PbreOR20
MVVPMSKIGYKEALAIPISVLTKMGLWPAKKVNLIVISKLFMNIVIAIITETVMVSNIIQAARS
VNMKLLNWSICVFFPLTNLHIKALSLWINRGYFLSLLNDLDSTSFNNHPVKLNRHIQTIAKI
SDVIVKYFALVMVIFLSIFSLLPSFTNLPLMMPPPYDMGKLDVAYRIGHLLATSYLATMSATID
SLYMSLIALSNAQLDILRERLINVSEDAKDLCDSMDKMKWDAGVWVILKECVLLHDTITKY
ISKLSAVLSLPLLFQYASGCFIMCNTIIQITILNERDSRTIIGMCGYSGIVFAQMSFYHWLGNEII
FKSDKIIEASYTSNWYELDIRSRKCILLLMERAKRTTAIKLYDLVFVSLESLGVVYYSYFQDGFS
STFHRCFLGGSMGIFSFCLS
>PbreOR21
MLLLGRRLLSYSCFMLSLVDANPFDPRKVTIKCKVMLVFEFALVLSGLMTLKDYRSVNEDL
WLTCFSASQGAVKVLGISVNKKGFHEIAKMLNYFQLRRKNGCSQAKRPLKKLVKWYQAIY
AFALGVGVVNYMTKPALLRQRILPSEGYVPCEISATWTCYIIAYTAHCLGGVYTIITLVSVDCI
FWTLLYYGYAEMEYIKICLSRLSINEKRNGDDPLVIQEIALLIEHHDKTFTYLKKINSVYAGILI
YQFIVTLITMGMAQYCITVDGFPPTLVLAWMDSPYIILGYYQIFGYCMAGGIITTQVFGIHCT
YMSENSRRFIFSSILKSQSIADAAFESNWSIKCQPKLRKSLCLVILRSQRLLRITVGNLWDLCP
ATFCSVVKASMTMLAFMKMMYGR
>PbreOR22
MENHLGDFQQKSVLEFNLRILQIFGLWGIENKLNRAHKVYRFVMVIFFSLHVFSQVTGVVQ
LARDNLLDALELIYIPALSTGLFIKVWYMVKYEKGLEDLINSMDEGIFKPRTTEQAEFVEKYT
HSMGVLFRTFIIASIFTWTLFYATTLKNIQQRSLPIAGAYPYKQELFFVVYYLFQGLEELLVAFC
NVSVHCIITGFLCHMRMQLHLLNENLKNIEALSVQKLDKRQGISPKNSVVREKLQDMMDE
TLVECVEHYGSIIKMKTDFEDTFGATIFFVMIVDCFVICMTLWQFIVLSFRSFKFFDVLVYLTC
VITELLLFCWPGNELILKSSEVPYSAYESEWIQRPLYYQKNLLLFMTITVQPIQITAGYFSLSIQT
FIYIMRAAWSYFTFLRQSFV
>PbreOR23
MSEKIAHQFMGVNVKILKFTELWLEDGGLVISKVKIIKLFVIFLMVCPTCVALLSECVIVFIDS
EPDIVHRIQIILAESCVLGAMYMVICFWKNRSQIKDLVDSIADFANYSELDLLIIDEKAGFFSK
IVLGYSLSGMVIYTLSPLLSTESCEKVKSQYKIDHGIPCGIIVPIRIPIDPENSHIFRFYVFDQVV
NGVLAVLVVVNITMMVCGLVEHAISQLKQVRCLMSKLSESGNIEEKLGFVVKYHYAVIRFIN

NINKYFGSQLVLHFTLTSVVISLLGFQILMVNDRKESLMYALHLIGWLIMLYNICYYGQLLID
ESIGVAFDAYSVPWYNCSVRVQKDIKFIVMRSQKPLTLKAMNLGIMSHPTFLGVISSSYSYFT
LLLKVKNA
>PbreOR24
MSEKIAHQFMGVNVKILKFTELWLEDGGLVISKVKIIKLFVIFLMVCPTCVALLSECVIVFIDS
EPDIVHRIQIILAESCVLGAMYMVICFWKNRSQIRDLVDSIGDFVNYSELDIVPVDKKVGFFT
AIVVGYSLLGMIIYTLSPLLLIESCEKTKSQYRVDHGIPCGVIVPIRIHVHPENPHMFRLYLAG
QVGNGIMAVLVVVNITMMVCGLVEHALFQLKQVRSLMSKLSESANIEERLGFVVKYHCVV
IKFINNINKYFGSQLVLHFTLTSVVISLLGFQILMVNDRKESLMYALHLIGWFIMLYNICYYG
QLLIDESIGVASDAYSVPWYNCSIRVQKDIKFIMMRSQKPLSLKAMNLGIMSHPTFLGVISSS
YSYFTLLLNVKSS
>PbreOR25
MTQKPADVIRFGRKALWLFGLYHGKNNEKYFFYYTIRTITIVVISMFPLLLLLKLILRPCDVHI
FLDSLMYLTTITWFCIKIYLHLYRLKKLRKLEDFVDSKILNLQTEEQARFVAGAMTKQKLVIS
TFRYMTYIFTAIFALYPIIMGKQDLIMPIWTPFESQMEELATYVFETFYLSYVIMFYPSLDAIYIG
ATQTLVSQFQILKDKLKRALDRSAWESTINENIETKRQLKICVAHHNAILELAYKTEDLFSPIL
FTQTLFNALEICVTGIQVAVVESSGVELLTTFLYLGIVMVQLMLVCWCGQDVITESEGLITAC
YMNQWYTYDTSTKKYLFILMERAKEPVALKAGPFFRLSFETFVKIIRTSYSYYAVLQQLYQK
>PbreOR26
MARISCTRTIEKPKRILSMSGVWPTAKITKYTVLNLIFNLSFDIIVLTVIILNILNALKFKKINLL
NKMICIVISMVNYIAKSSTLTINKGCFMSIIQDLESTTFNRHAMELNYHIESIYNNCNLVFKYF
AFMLVIYSLIGSILPMIVDIGITIPAPFYTGRYEFLYKVLHFFATGLLSCNTIGLDILCLTLISLGIA
QMKILQESLENIVEDREEANENTYFLNLKVDSTLRECAILHQKIIVFVGKLNTLLSFPLFIQYT
CGCFILCNTVVQLTLLREPGTSNTVSMIGYGAITFGQMAMYHWFGNEMIFTSEKIIESAYLSK
WYEIDVSSQKTILLLMERAKITQSIQVYKYIFISLDSLGVIVRWAYSVFALVKARYG
>PbreOR27
MFQKILTEEVFFLLNIRGQSLDASTLSSKLKAIVCLSLEGLLGILLTVGILDSTDDLYSVLENIT
GLITLLQIIFRSVILFVYKDNFLNSIIFLKIFWKADRFGATSFKKISNFRLQFVKILRIYRLIIGISG
TLYLTKPLFEKHRMLPMTCLLLCDIHNDVCYVFYYIFQIIALATQLIMLVGFDSLFFVLLMCA
YVELEQIKQALSNLSIKENAKGDQDKVLKEMITIIEHHNLVLGYIGIFNHLFKTALLFQFGFSI
FSLCSSLFVMTTNGFPPTTGNFLKSGPYCFSGICQIFIYSAVGELIAQQTERISDAAYETKWILN
YQPSFRKMLLLLIQRAQLRSQITVGGVWKLDMTTFASILKGSMSLMAFIQTVYYNKK
>PbreOR28
MSKRINQYDSLDFERKVLYLTGVYCGRKFPNNAAFKIYRIVNTSTAFLFLFLMAAKMIVERD
NLELVFDVLHTFISQITLIIKAIYLCKVLPKYNALEERLTEPIFNQQTPDQDALISENILGYKYA
AFTLHGLAWVALVLYTICPIIEGPILAIPFWLPSGLDNYKMFVKIYEVLCTWTLSSGDPALDLL
PVCLLSIGTAQLDILNQNLTCSADRDPDDSYEVQEKKIRVRLKNCVQHHLAIIKYLDDVQDI
FSFGMFIQIFTSVVAICMTGLQFISITPDSTVFFQVVTYFGCMLSQIYNVCWIGQKVITKSDEIR
DACYMSAWETCCSSNKKTIFIFMERSKRVMMFRAGNFFTLSLATFVLIIRNAYSYFAVLVRVY
K
>PbreOR29
MVRRPQPLDFTKFPRRVLWFFGFYFSDDMQKHMFHRAVCVAWLALITPFPLMILLKLLVEN
QDFLSLLETLHYLFLHFWIVVKVFLLLKRLPKLRRLESWIQSEIFNSHDQRQDRMMSEALRK
QIGFFKLLWSSTMSFATIFAAFLPSDKVSLNIPIWTPIKLNRMIWHVYEVLCYIVTASAYPAID

CVITGFIANMTAQLQILRCNLEKATFRNSGDDYAFTERKIHQRLRRCIIHHNAILEYIDETEH
TFSLSLFCQILFSVLGICLSGFQFLLVEPGSEKFILASVYLIIMLFQIYFICYFVEDMVMEGFGIISS
CYTSEWYTCTTSTKKLFFIIMQRAAKPITFRAGYFFLLCLYTFVNILRNSYSYFAILRQVYRD
>PbreOR30
MEDEYVMKCGLYYLHLIGTNPFKRSIRGNIICFIQTFTAIYSLGMAIDGVISKYENIKSLAGSID
TVPSAIQTVIKLLSAIFLRQKMRNLYLMVEEKWPDDIFGREFQNKLQKWSKMFKTSYKVYLY
FYVFTLVFFISRPLLRHSRILVTEWNLPCNVASNQCYGFYVVLQASYFSIIVSVVFGFDIIFYAFL
FYGFCELEKIKYAFEHLSISEDVEGDEEKVYVEFCKILKHHDYILRFLDMVSKVYSLQLLSNFS
AYVSTIVFGVFFMNVDGFPPSKENLAKYIPYLITHHFQLIMYCVLGEMIRTQLSSVSDTIYLSK
WYVKKQPKLTKAMIIVMQMSRIPNKILIGGIWAMDLDLFMKVVKTSISVHAFMQTIYQVD
>PbreOR31
MAKMNCIEPLRRVSNVLLREGLYPNEKIENLQLVKIILHFGFYTLILSCVIMNGMEAVETGN
AMALNHATCIFLPTFNLAIKAMAVLRQRKHFLSLMEDLKSDVFNSHSEKLNEYVKFVHRT
TELILRYYTIVMSAYVSITSVLPFVTNAKMMIPPPFDMKQYVVIYNIFHVFIGAYLALNAASF
DVLFMSLLGSCIAQLYILEQRLITIYEESRVKSKDQDSIPLPLREAIIVKQCAVLYLKISQFSEKL
CILLSLPIIVQYAFGGFIICNTILQLTIMPEVNTGGTIGLLAYSAITFSEMAVYHWLGNEILFKSE
NIINACYLSKWYKLSTKNQKMLITLMERAKRPLVIQLYKFVFVSLESLGVVIRWSYSLFALIK
>PbreOR32
MTIIGRVLIEYPCSVLSLYGVDPFSREKTPLKCVAMLVFQFLLVFSGIISLKDFQSVDPDLWLTC
TSASQGAVKVLLILLQKEKFIELGDMLNTFLLYRQQQKDTEDDKKIEQLGKRLQKAYAYALA
VVLICFMTKPILVRQRILPSVGYVPCDIRATLACYLICYASHCFGGIYTVTCFISTDSLFWTFLC
YGYLEIKYVKHNLLNLKINKGRGGDDPEVLEKISSLVQHHAQILTYLQKVNDTFAAMLVYQ
FVASLCTLGMALFCLTMEGFPPSLRITVMYSPYYLASAQQIFVYCLAGGIISNQSASVADAAY
NSEWWSKHQPNTRKALSLIILRSQREVKITVGGLWILNLNTFCAIVKAAMTMLTFMKNLYG
RE
>PbreOR33
MGTIQEIPMTEVAIFLLELIGEVGQRSSASIMIFRVFNCSVFVIIIWYAFANLFVVTGESFVNSLL
SVLYASHGILKYFLLIHYKPIIESMLKDIGSKFWDHRDFSINIAETTEGIFRKVNFVQKAIVGG
VLIVMYSYHLAPLFDSNRLFLFETKAPESPFMNAVLLMSQYYCVSMEVPIVLGYDCIYFTICI
HLVLQLRLLKQKIANLLETKEENVESEIYKCIRYHQFLISMFTRMKDTYSAMLLFHYFDTMIT
TCSVLYEVVDGGYTTILNFIGKLITMIFFYAQFACYTFPADQVAEEFSGLSHSIYNSFWYQNTI
AVQKTLLFMMMRAQRTHYFSGLGLIDVNVDAFGSVVRKSFSFCTMLRNFVNKEVEV
>PbreOR34
MHSIQRKMLEPQHTIEQLSRLISKSMLTFKESATLLFECIFPLLVTLCWIFEDLSSYVLMESITG
GIPAYQGVIKKLVLHSYKNKIREIMESLKKFWPKNKFGKAQLVKVNAYEAKIIKMLKIYSMV
LWIGYVAFVSMPLVQTKNLPVTWVTFCDIENNIYCYIFNYVLQSLCIAKLMHLLLSLDTLLF
VLLWHGYCELEKIKYGLAHLGENDFTETVAVKDMASLVKQHSLVLIYIQKIFDLFSNLLLFQ
LLTSSTGLCACLFVLKAEDEASIVAALTYVPYLCGCLLQAFLYCGIGQTIYRQTSSVADAAYA
SNWVQKNAPTQRKALCFIIQRAQKPEQMTAGGVFALNMETFISMVKGALSVVAFLQAVY
HSK
>PbreOR35
MTIIGRRLLHVPRALLAVFAIDPFRQEKMSLRCIVLDVFQFYVTLTGILLMKDFQNVNADLW
ITCISCSQAAMKVFSMLTQRRDFFEIGKTLNYLQLHRKYSNSTDGKYEKIGIQCHKAYLFTIV
VTVINFMIKPMLIQQRILPSVAYSPCDIQYSLTCYLVNYICQCCAGIYAALMISAIDLVFFSLLF

YGYFELEYVKINLLNLKVDKKYSGDDPKVLKDIAFIVEHHDQTFRYLELINKAYAGILIYQCC
STLFIVGMSQFCLTINGFPPSMNTLLAYLPYYLASLGQVFIYCIAGGVIADQSESVGDAAYASE
WWIRNQLKMRRALSLIIMRSQRRLNITVGGIWVLNLPTFCSIVKTSMSLLAFMKTAYDN
>PbreOR36
MSWLSELDIFSVGFNMWKFIGEDVYLTSKKLILFRIINCTVMLTAIIFILSNLAKAEGEVYVET
LENFISVFHVLFKYLLTMYYKKDFAELMQKKAKHFWRNSRNGDKHLTASIDNLYKGINILQ
ITMAVGLIVIAEIYFLIPYFNPNTIYFFHSHVFVDSVIVEVYLLACQYYTILLVPPIIMGYDYMY
LCLCTELAVQIKLLKQKLKETFTSTNGDILKSIAICVEQHELLLWIHNRMQRIYSITLLFHYFV
TLITICFDMYQAFVRQNDLSNEILKMVSLTAIVAQFAFYCVPAELVSSEFADIANAVYMSKW
YNHKAKIQKLILPTMIRSQRSHYLSGLGFVDINIEAFGSVIRKAFSFYAVIKQVLNE
>PbreOR37
MLRVNCIQPLQKPMRILKVNGLWPLVIVTKGVEFKYCCNFIFYVLLLYIVLHNIVNAVVAKN
INLLNWMTCVLMPLINYYAKAITLFANKTYLISILDDISGDIFNHHSQKLNRHIQFIYRVSNI
MIGYFAFVLTFFILIFGVLPIVTNVRPIIPAPYDLGKYGILYNIGHLFAVVFMGAISTAFDVLFM
SLMALCGAQLDILEERLMNIWDDANAAFEDPIFKQRFAAVHDVVQHILKECVILHETINGF
VNKLSILLSFPLSIQYGIGCFIICNTILQVTILSSPDSTNIIGLCGYSGIVFAQMAVYHWLGNEIL
YKSNKIIEASYLSEWYKLDIRSQKCLALLMERAKRPLVITVYKVIFISLESLVQVN
>PbreOR38
MSWLSELDIFIFGLTLLLFIGEYMEYTPKEIKVFRVINCVVIITVLVFILGNLADAKSGEFVSTM
EDFTTVFHILFKYLMFMYNKNNLKALMVAKMERFWKIGPKDTDLSRKIHNIYKGVNLLQI
VMLADIVPIMGIYVFSPYFNPSKVFLFAANVFVNSVIVEVFVLFCQYYFVIFMTFISVGYDFM
YLSLCIELRMQVKLLKYKLTGVLTKTNGDDVVFGIRICVKQHNFLLSIHDRMQKIYSSTFLFH
YFVSLITACFDMYQTFIGESDLLHEVQKFSSLAAIIAELAFYCVPAELLSAEFTDIAQALYMSK
WYKHRPDVQKLMLPVMVKCQRPHFFSGAGLFVINIDAFGSVIRRAFSFCAVIRNVLDK
>PbreOR39
MQRQMKDEILHTGITVMGFIGENSSVATKKLLAFRIFNCCLLVYLLVFVFANFSQAEYGMYV
KNVQSAVSVFHMLIKYLLFIYFKLDLENLFNDKLNFWDYRKFDVGTIKRTATTYSIINIAQNS
TIIAGAVIIVLYYLKPAFNSNDTYIIDAWIFVDYIIIDVTVLACQYYFIAAITVVVIGYDSIYLSFC
THVFLQVKLLKCKLEELPKYSYKTAEREIKMCIEHHQLLISVFKRMQRIYSIMLLFHYFITLAT
GCIDLYELLIRKSNLSYTVIMVISLLFTYAQFGYYTIPAEAVATEFSDLSTSIYMTNWYDNTVRI
QKLLLFMMMKSQRQEYFSAGGMIDMNIYTYGSVIRKTVSFYTILRTVFNK
>PbreOR40
MHMIGRLLKSPYFLLKTVGLNPFDPKRFGFRSIILMVAQIVMAVLILMGLKDFRSVTADVWV
SCIMALQGVIKGSSAILQKDAFYNIGEALNCFLSRKFTSSEQKIEEFVHRMQYTYTITLILALV
AFIIKPAVVKQHILPSAWYDVCDIEATITCYLLCYGYQCYASAYCVIIITAFDCMFSGLLAYGYI
EMEYIKRSLLQLNDNENCTGDNQEVLLNIGILVERHNQVLRYFRDIDKAYSGILMCQYAAT
LMIICSAIFCLTTSGNQPPVDVIVTSVSYFLAGITEIFFLCLSGDIISLQSESIVHAAYGCNWNH
KPKLTKALCLMIIRSQRACRITVGGLWILNLETFTSVMKATMTLLTFMKKMYGR
>PbreOR41
MKQIPRDDIIQMGLALLKIMGERMHFKSTLLNCFRTFNITMMVVNLFFVLAYYPQIGADYA
KYIKSTECALTIIHVIIKYSLFIYHKRNIEDLLDHLLEFWDYNSYGDNIRLTTSTLFRNVKVGQ
FYYSIVTLACIALVFLKPYVNPDNRFLFVCWTFSGSTELETTVLACQYYFWSILYPIVLGYDSV
YFSYSMHIIVQVRLLKQRLQNIPSNVHIEEIVTCINHHKLLISIFARMGIIYFWMLLLHYFITLV
TGCSLLYVILLGAADNADLFATIFYLIGLFIQFAYYSFPVEEIVFELTDISRAIYMSNWYEQNVK

IKKILLFMMMKSQRQNYLSAGGIIDINVDAFGSVCRKAFSFYALLKTVIDR
>PbreOR42
MLVYCHSHVTSIDTLAALFSQMAIELQVMCKITVLFIKSNRLSNLINLVWYEFWPSNILGNQ
LERKIWNDTRRVMIPIFLEIIMSIIYSSICLFLPLLLSNKTPILLQEAWYPLGWDRNFISKFVHVY
KTVVAIYTVTGMVCSYDFLYITLCVNCAAQFHLLCGAIEFIGSGKDADIIKKIQPESNVIKDSS
LFREENKDEIDLLTICVKHHRKLIQFGNELNVIFGSGHFLQLCNTLMGICLTTYTISSGSNSFI
QVILNILYYAAHFWQLYVFCAASSYISDSSATVFTAAYHSKWYTQSYYNIRKYLTIIMIRSIKPI
TMNAFGLFQLDYALFLTADYEMISKVVRVVSALIFFRLCVFHSLYTLS
>PbreOR43
MLEEMAKQLKAECLPSDASKGTLKLKFIASFVFDTIYPILVTTGWFFNAIDVFEFVDSFTGAL
AMYQAYVKMVMLYVCRKKIVNLFDCTGEFWPADKFGKKSLKRIDIIQRKTVQIIKAYNVLL
RGAVFVYLLKPLFDRKKVLPVAWYNFCPFEDNKCYFSNYILQAIYISRYVQELIRFDNVFFLF
LSYSYCELEKVKYGLLHLNVRHPHVLRQIAALVRQHNRALELIEKINDLYGNLLLFQFSTTLI
GLGSSFFLLTTNGISLISRYIPFIASAVVQCFIYCAIGQIIYEQTISVADTAYSSDWWKVNQPQLR
KAICLIIQRAQRAVQITAGDMFAINIATFVSIMKSSLSALTFMQTCYHPE
>PbreOR44
MLPANYMSQVPLSILKFIGEHMDYSSKLLNLYRLFNFVVILTTAFFVICAPFNKEKLTRYAEY
LESSAVTVQVGIKYLFFVYNKSRIRNLLAELAKIPWDSKDFTRNFLTSDQKFQKLIRLAALSF
TIHSTIVVLVFLLRPAYQKYAYILETLDVDSIILSTMVLALQYYILMLVPAVVMTCDCLYLALC
AEIMIHLRRLRHRMERLFENIDDTSGVRNEFCELIKWHEHLLRVFSRLQEIYSFMLLFHYYTT
LQTNCAELYTFLGSAKFEIKYLISVIAIVSEFALYTFPAEAITVEFEKVSTSLYMSDWYKSSLDC
QKLLLFTIMRAQVRECLLGGGIVEVNLRAFTSVLKLSFSLYTLLRNI
>PbreOR45
MSLYGLNLFNPSKNYRKFLIIWLAQTVIASTTLVVLKDFQELDADVWITFMAASQGTIKSVV
LLIKKETFVEVKELLNNLRLQRGNVEDTDSMRNFECFAIRCQNTYSFLQLLSLLAFMGKPFV
MDGRILPSEGYTPCDIRGDACYIFSYILQCYAGIYSILVIVSADDLFWGLLSCGYIDMEYVKHI
LLHLKVEERVEGDDSKVLQQIAFVVEQHNQILMYLKKINRAYAGLLVYQCFITFFIVGMSQF
CLTASGFPPPLHIVSTYAPYFLASLFQIFLYCMAGDIVARQSETIGEAAYGSKWWLKHQPKLR
RALCLIIMRSQRTLKFTMVVWVLNLETFCAILKTTMSFLAVMKTFYE
>PbreOR46
MFAYFFIFVRDVEALAVVLPSLSVGMQVIGKTAILYCKTKKLEELIKTVWNDFWPSNLMGEK
IENQLAQDTKRLLTPIFVEITIGLTYSAYCIIVSLNNREPVYPAWYSFDLSKSPAYVTVAILQIFL
TMYVDTCLICGYDFLYNAFCINCAAQFRLLCHTTEYIGSEEEYRIIERIQNYANVKSARTLNS
VGDQEGVLLRICAEHHQKLIRFCNELNQIFGHGHFLQLASNLMGICTSCYRISLESNYNNIL
ISVTHYITYLCQLFCYCCASDHLSNWSTRLSDAVYHSSWYIKKCLIKGKCLSIMMIRSQKALV
MNAFGLFELNYVSFVMVMRFSFSLYTFLKTLSSN
>PbreOR47
MIDLNVWFLKLSGLWCILKSTKIARKYVISAAFIIIPSQGIYLPMECYKLITGYENFQRTLELLG
FALTHILATTKIINLYVNRKEICQIIEDLNSYYIMSDHTAAEECKRLKQRFYKKTYRLGILLFVL
GNCAGLIRFGISAFHLVFCTDDTKYEELCRTMQPFLIPLPEFLDMIFLRWVLCVFQGICLNLY
AWQIVACDMLFVSLLVHIDCVTHILCYLFETVTERSMSHRNTISDKDSGQQLNSRMNKEIN
MATYRLQRLITTCEKTSNVYQYVVLMQLVFALFTLMSSLYVATSVPIFGASFIYQLEFYVTIVT
QLSLYCWFANEITISFGQIPKAIYNNNWMSG
>PbreOR48

MSMVILKDFRTVNPDIWITTVAGSQSSIKMFVILTQKQKFFEIGEIVNNLDLRRNCEQAFTKE
QKDFKRFFANLQLIYIFIIITGLTALAVKPIVLKERSLPSPWYPVCDIQTSITCYVTCYMILCITGI
ILVIILLSVDLLFWSMLAYAYLEMECIKYKLLHLRIDKNASGDDANSLSQIASCVKHHNQVLT
LIEKINHSYSGMLIYQCLVTLFVVGMSQFCLTATGSTPSLPIMLAYIPPYLAGFIQMFGYCFFG
NIIIEQSKSIGDAAYNADWWAKSQPKFTKAIMLIILRSQKPVEVTVGSLAVLNLETFCAIMKT
TMSMLAFMKTVYNA
>PbreOR49
MVSMIGRIALELDNIPLILEALYFGLTQTTFLCKIFNILVCNYKMRRLVDYLKNPLFNQHTKE
QDIFIERAIRICHIFANTYRVCVGLTITFYAIFPFIEHTLPLPGWFPMDSKKYHYVLYIYHLACLI
INGYPHTSLDCINASMISMASAQFEVLKDNLNHLIKPNDMKLTIKKQDEIIKKRISKCIIHHN
MIIKFVSHIENMYSNVIFIQFLCSIIIICVTGFQVFVMSPSRIQYITLVLYFLCNLTQIAIYSWFGH
DILAKSGGIGQACYMSEWNNVSPNTRKLLFIIMERSKIPIILTAGKFFNLSLNTLMMILRTSYS
YLAVLQQMYKT
>PbreOR50
MLVKTFESAITILHILVKYLSFMYGKIGLKAIIEARAKFWDPEATNEQAAQFAKKLYSSTKTL
NTTLLVVFTISQEFYWLKPIYNPGEKFMLNARVPYNFLPLEAMALFMQYYSVGIVTPTVMT
HDAMFLAICAHLSVQLRLLRCKIYEAAAGEWEDLKKCIEYHQFLSRIFIQMQEIYSVFLLTQ
YFISLGILCVQLYILNSRALNIADTIELLLYLATMYCEVAFYSIPIEDISSKFQDVGNAIYMSEW
YEKDLVRRKNLTIMIMYSQATRYLKGGSIIPVNVDMLGSIFRKSFSLYVLLKNVL
>PbreOR51
MLVIHVLLKYFLYIYYKPDIKQLLQDKFEEFWDYKRIDNHILSGIMKIYKAIKLVQASVIAAGI
AAYYLYIFRPILTNGNPFPFDAWVSGVMILDILELAGQCYYLCLTVPVVVAFDAIYLCLCART
AGQVKLLKNRITYFQELVVLDMGHYISCHQFLLSTFAHLRRLYSSMLLFQYIMTLITACTSIY
ELLMNTTNTNETVNNTIVIWVILLQFAYYTFPAEMVAFELSDLSNAIYLSKWYLHNIDVQKQ
LLFMMIRSQHQSYLIGGGLIDINVRTFGSVIRTSFSFYTVLKNILAK
>PbreOR52
MNICVVTVCHGACLFKATTEISVIISVAATFNALIETLVRFHILLTKKKILNEILVKVWKKFWP
AQAILRPEIRNKLRKRVIITFVLSIIFLITGTISAAEITTVPYLKERELELRLNVPFDWTRKFLYEII
YVYQYFIVWYSFSMINTFDIFFVALVNVCIAQFVILQEALKFILSEESKRQRRIIFGGKAENMT
DSDMLSECLKQHVLLISICNDMEKSLNRAILMQFIVSATASCAGFLIMKIDYNQFSKMLFYA
VAHLIQLFHYCYVGENLSNESERISDVAYECNWH
>PbreOR53
MADKKDISYKRLLSKTLSYLGIVGLNPLAHTKASDIKAVVILTLEVLLTFSIAASFSINLSEPYA
IIENFTGFAPAVQIVYRKIMLLMRRSDFRDIINYLDYFWSINAFGKHAANQVIRIQKMQSKFL
KFYLWTVILSGIAYFDKLNKLFSLMLLFQFSSSLCSLCTGLFMLTSNRSPPSSSFALKFVSYVISA
LIQIFAYCLAGQMILEQNESITASAYFNCKWWEAYQPKLRKGICLIIQRSQRPVNIVAGGLWK
LEMDMFVKIVKGSFSLLTFMQTLYIPK
>PbreOR54
MRKLSFEFSRDIYKDGVARCILPGKIMLEGIVCWPNKDTLFMRMLGWFLFYNLLLVEIFHAA
YVFKNFTNIGEAVSAGTTVTSTMEGLVRIYTMLTKREVINRILVKVWKQFWPIDVMDPTKQ
KEAKRQSYISIALTSLVLLVSGTCTVFITSVPYLTNRGLVVKSVFPFDWSKSYVYELIYIWQHFN
EWLVIVAINGFDFFFIALVTICYIQFIVMQQIFRSILTEDSRRQRVIIFGKRGETMTDREMLFECL
EHHNLLTGICSELEESFNIAILIQFFVST
>PbreOR55

MWIGVLTVIPYYIHPILQEASANEATYMNVTHNNITKLLKIRPLPLSSWFPYNRYEYYYYSYA
YDIVAAAIGASMVVLTDLLFVSIMIFLIGQLKTLQYHFKNAKKIAMVLKLNIGTTYNKSLNY
TIKYGIRMHQFIIRYVEDLDKSMSRLMLVDFAVASLQMATLGLQMIVVGVGWQQCFALEFLI
AMLIQLFLFYWHANEIMLQSMEVGNAIWESDWLEYSDELKRSLILVMLRCQKPLTLSVGPF
YHMSTQTALSILRAAYSYVAVIRQMYFRKG
>PbreOR56
MKMPVEIRMRKLSFDFSKDLYKDGVKRTILPAKIILQSICCWPDSDTAFALFVGWLLFANLFL
ILIFHAAYVFKHFNDIGDTIAVGVTVTTTIEALARLYLIITRRRIINSTLVMVWKQFWPVNALE
TKATQRIERSAQTVVILATMLLILASSCNCFLSGVPLVRYHDIVFKSAFPFDWEMMYVYEFIY
VWQYFISWFEVLLVCGFDFFFTTLVSICALQFTIMQEIFKSILTRKSKKQRVAIFGERGRKMTD
REMLFECLEQHKLLIG
>PbreOR57
MEFQQVNFQQRSVLNFNLNVIKMFGYWQPENLPKILRCFYRIYSFVIVIFAIFHVVTESIDIIG
LVTAMDIDAFASNAPLYFLNFSYVIRAIHFHAFKKEIISLIDAMDEHFFQPRTEDDMTFSIRH
AKKYVMFCKIYIFCGAATSLLFVIFPFTDAEELKLPFCGWYPVGDDWFAVLYVYQGVQEFSV
GMCNISFTCITVCFLSYLSMQMDLLDTCVKHLKERCEENLKKKNTDLLNVTYEEKLQKAM
NESLIDCISLHQ
>PbreOR58
MGETYNKNTLKRIIFFRIFNSVIMVYTSLFITANFFYASGGNHVETMQGLILVTHTLLKYLLYI
YFKPGIRELLQNKHEESWAYQDIDNHIMAAIIKMSKTTELIQVFVLLAMSITAILYLFRPILTR
NNPFPFEAWVSGVIIMDVITLACLYYFFCIVIPIVLSYDSIYLCLCIQTVGQVKLLKNRLRYFQ
GSVKADISYYIKYHQFLLSTFSGLRRLYSSMLLFHYFVTLITACTGIFEVLMGRNSATDIAA
>PbreOR59
MSPKIVIRKLSTEFSRDVFKDGAKKVILPGKILLQGVCCWPDNQSAFMNIVGWSLFWNLFIV
EIFHTFYVFKNFRDIGDAVSAGATVTTTMEGLVRLHIMLSKREVLNGILVKLWKQFWSVEVI
EPIKCKKAQRKAQLSIVLTSIVLVSSVISNTQISGAPYVRNRGLVLKSVFPFDWEETYIYELVYI
WQYYSDWFVLFMINAFDFFFIALVTICSVQFVIMQEVFRSILAVDSRRQRVLIFGARG
>PbreOR60
MSRLPEGDILEFALQVYATFGENLSNTPKKIYVFRAINVVMLSLTVIFILEACIEEEGIVLVKTL
ESAITVLHVLIKYLSFMYSKEGLKAIVEARLKFWNPQAINEHVAQFAKKLYSYIKTLNTILLV
VFITSSEFYWLKPYFNSGEKFMLNARVPYNFLPLEAVALLMQYHSLGLITPIVMTHDALFLAI
CGHLSVQLRLLKCKIYEAAVGEWKDLKTCIEYHQFLSRIFIQMQEIYSWFLLTQ
>PbreOR61
MSNIRRPKPLDITYPARKTLWFLGLYTGRELPKHILHDLNYTVVIAILALFPNLVLIELLQHD
HSLSDILEILHYLLVHIWMVIKLMLYLSQRSRLRILEDQLESVDLNVQNKRQDHFISSAIGNQ
KFFAGFLLVMGWIFTLMFATLPLVSEQSLLVPIWTPMELNEKICHAYETIYYCVMVCIYTSVD
SMVIGYTASVTAQLEILRDNLISVAERDEGLNQTVQELVIRKRLKNCVIHHVSILK
>PbreOR62
MLLVISVCLAVLGLLPYGVTTATRITFSRQHYVFVFYDILDKGYTFGSSNFTEKILVTGDAIGS
VNFALDDTRYLDIEPVASEKNGEYYVTLKVLNTIKAPFRSIFRIIAWDARKVATARVELQIKET
YFATFLQPVYYGGVTEDLKVIIYDGAIVLNGGDAKDYHVAVAKEYKDFFDIQFSSEDVELAV
RPGQTLETLPKFPINVTLTAYDRYLNKTAETTVVLNGPLLQRPKGIKPLLLW
>PbreOR63
MTLNGRRLLDLPCAILAIFGIGLFSPAKISLWCLFLLLIQFLVFITALVAFKDLRNMDADIWVT

GAAVSQVNKQRVGDDWEVLEQISFVVEHHAQILSFVQLINDAYSGLMIYQCACTFFIVGMS
QFCLTINGFPPTLTVMATYVPYYVAGLCQMFTYCIAGDIITNQAKSIGDAAYNSHWWIKHQ
PKLTKALCLVIQRSQRTLTVTVGGLWVLNLETYSSIIKTTMTMLAFVKTIYEN
>PbreOR64
MKYLAFICYKPDIENILCYQRKAFWLHQDFAENIAIPCNDLFRKIKVFQVVVVAGTYTAVLL
WVSRPFFSNTLPLEMWMIKEGSGFSALIVLLQYYIFAIIVPIVLGYDLLYASFCVEVIVQLRLLK
SKLKYFGTNKVSDASNELQKCVKHHQLLLDIFKQMRDIYSIMFLFHYFFTLVASCVELYELIL
GHLKVSRIFGNLMNIAMLLNQF
>PbreOR65
MQRKIIFDDRAWKMTDNEMLWECFKLHQLLIDICDAMEATLNRAILIQFAVCTSANCCAF
LVMKVDYQQFIKMLFAAVAYLFQLFYYCYVGQQLSDESEKLTDAIYECDWYLEYNRDFRKG
MVLMIQRGQRKICLTAVGLFELDFSSFVKILRLSFSFYTLLDSLVMEKE

GR
>PbreGR1
MDTDHGYHVRNPHQKITENNIRVERLSNAGLETISHRGDEDEPDPELLERFDSFYTTTKSLL
VLFQIMGVMPIERASNGNTTFRWFSATAIYSYILYIAETIFVSIVFKERLLLILQKGKRFDEYIYS
IIFLSILIPHFLLPIAAWTNGHEVAKFKNMWTKFQLKYYRITGTAIIFHNLTVITYSLCIFSWVL
GIAIMLAQYYLQPDMQLWHTFGYYHILAMLNCLCSLWFVNCTAKGRVAKDLAQNLHNA
LESADPANKLAEYRDLWVDLSHIMQQLGKAYSGMYGMYCILILLTTIVASYGCLTEILDHGL
SFKEAGLFLIAFYCLSLLYVICNEAHHTSSKMGPEFRDRLLNVNLMAVDYRTRQEVHMFLT
AIDKNPPIMNLNQYADIDRRLITATVTSMATYLVMLMQFRSTLMRNAALAARRSAMNLNR
TATNATT
>PbreGR2
MKFCWKSKQKDSTESFSYWKTIMQSLEEPANVYDTLIPVHVLMKSVGLSPCILVVENGKLR
YRSSTLGSIYSVLAILLFVGYYIYAVEERETNDETNKVVRSIDMYHLYGSMIVMACCIILNSVH
QKTLIEAIDRLNDADVIMAGYTKKIDWKTSRNNMTVYLLVIMAMLAAGEFMNCTMFLRQ
VATLNTYCLLMCYIPMLINGFVEAQFVCHILLLKQRFAVLNGELRRLITKQRPLPSFKIIKVGP
PIVEEEPKTLVPSKLIHNARQLHCQLCEIGVLLNRYYSKQILLDIGDVFIGFTTLAYYCFDGC
MKLYMKDDESNLYNTVTTGVWTLVKLSRLLVMTLSCSIVKNEAKSSGDIIYKIENKYQPELS
PEVYAFGKQIIHWNFKFTAFDFFEVDMSLFYSAVSSATTYLMILLQLDIANKQIEKSMENSEN
HSH>PbreGR3
MVATFMVKPSRDTNIRYITPLFKLCTLLCIIPPYDFRNYKFTICIKYRIYRALVLVFLLGGAVYC
FISKVLYTFDMIQNTVVVVNVLAYISFFLINVTSVTSANFNNFKSLDKFMNELVSIDRKLLAY
HITNKTKTSTWLWLEIFLNHVVLFFVMLFDGTLWYLASDNKFHFYMLYENLQKYHTHILSH
LMVNLIICLKHRYRIANNLLAEAVKQRDVVNISTKILFKPYKDVYTVKNVIKIFIGLNRMIEY
FNKIYGWTLLCIHINIIASLLVSLDFIIEFSSESNILRDKYGLEFILLMSMWSLMALCLGIFLAST
ANSTINEAQNTSNVSYKLLQYVPPSSTNPHDRELREDLLLLSEQSSLRTPCFTAAGFFNVDY
TMLFTLLSSITSYLVVLIQFSN
>PbreGR4
MFCFEMYGRINTNFINIVHSSTRTIVVGTINTTTDFLCNIIITLPAICSNSKFENTITRELEEIDIIL
SKYNTRRKKNRSPCTHLIALNLFYLGLVLNDVTVWFNSDYKNYHQQFYIFDQFYRYRASIL
VIYIYYLVKGFLRRITSINDTLERTFEHVSLDDIQHGTQTINNLEETAADISLLHAKLSNVIRSF
NEIFGWQMLCVLLNYILLFMVTYEMGLRIASRHLQNINWQFTVWFICSMVYAAIAVSAITSI

CDETTFEARRTTDLSYSYLYNLNLSKQHKEQASLVRILTTLVRQTRSDASITFSAAGFFYIDYS
>PbreGR5
MQYWQDTEVFIRCKIILPNRYSYVLFLIIGAAIVEHTLLITYSMTAEEYKKYQTNNTTRFEHFL
ARDSHIFKHISYTWYMGTFVKVISLANTFYWNFMDVFIIAMSIALAAMLKLINYRIRAARQV
RAIIWTDLRRNYDRICELSFRVEDIMSPLILTSFASNLYFILVQLHGSVKKRRATMESVYFFFSF
GFLLIRTLAVCLFAANIDDEHQTSVKLLSCLQSYNAEVIIFKSNY
>PbreGR6
MKDVNKMSKITFKESLKPLLVLGSFLGVISFNLTELTRSNLRIVTNVLLVIFYIILTGHSLYERSL
LDTTLLLTTTDMMQVSTSTLQIVVSWVMSALIQNKMISFLKRVSEIDKAFRQLGVYIYYDAV
HRTVIKRLLVRLTLGFASIISQLFIYDYALAISQIGFNVTLYFAILINVVIVEQFYTFAKLLRVRFE
ILNQHIQQVQKCVDVTKENISYIKLTGPIGSKLSTLRIICP
>PbreGR7
MLLMSFQASFVTIIVSLYDCSVYLRFFKMENVKELCGSIIMCCTYVLDCLCICYSCHSTVEAA
SKSGKLLHQIDTEDMDVKDQIEMFSLQIVNEKLEFTAAGFFTVNYGLLFSIIGGITTYLIILIQF
SADPSESKQ
>PbreGR8
MITNITIAIYGFTSEIVDHGFRFTFKEMGLLVDASYCMVLLYVFCDCSHNASINIANRVQTTL
LNIELSTVDLQTVKEIELFLIAIQMNPPKVSLRGYTVVNRELVTSSISTMAIYLIVLLQFKISLVK
ETKTT
>PbreGR9
MYKEQLGLDFQKFLKMLASGTVLQITIFLIYAIPTEMYLAEVSKFKNILFQETNKTREDGVRK
EFNSLSLQTLHERYEITAAGFFTIDFKLIFQILSGITIYGIVLIQFNPNARIFLRFMDSY
>PbreGR10
MYQFLLLVLQLAILGVHRTECSPAVAREQLIHENPGYIPVYIRVGDTPLEEINEDLAAAFRSY
AYKHYKLDYGDVKSGTQERRRKAEDNLILSSKMRKIE

IR
>PbreIR1
MRTLLVLLTAIVSVKSYLDDCSVGLLQDDDDQIKKIAFMAESEILGFENMIEPVTAFRSYDTL
TVTCHMIDSGVIALFGPQSEDNAQIVESVCNNKDIPHMETRWNDQPGKLTNTLNLYPHGP
TFSKALADLVEARQWDTFTVFYEDNESLARVSEILKRERELSISIKQLDAMGTGNYRPILKEA
WRSGHTRFVIDCKVDNLIQLLQQAQQVGMMTSRYYYIITNPDLQTVDLEPYKYSDTNITGV
RIIDPEAEATKRLVGMINQKQIDLELDPSHAQIIAPHELRVETALTIDGLNLLRMSLDHLPEH
TRCGPEIACQDKKGWIHGSTALNYMKMTSFDGITGLVKLDSEGHRTDFNLDIIELSEDGLVK
VATWNLTDGIKFLVGDDDYDLHDLSFVVITALTEPYGMLTDSQVSLVGNARYEGFAIDLIHE
LSLLEKFNYTFIIREDKSNGSKNKVTGKWDGMIGDLIDKKADLAITDLTITSEREEAVDFTTP
FMNLGISILYQKPQKAPPNFFSFAEPFAFEVWLWLGGAYFIVSISLFIMGRLCPSEWTNPYPCV
EEPEFLINQFSLRNSFWFTIGSLMQQGTEIAPIAYATRMTAGIWWFFTLIMVSSYTANLAAFL
ATENPDIPFNDVYELVEKASKSNIKYGAKNKGATMNFFRDSNNDDFKKIYNYMIANEKQV
MVGDNKEGVLRAEREPYAFFMESVSIEYEIQRHCNLSKVGDLLDEKGYGIAMRKDSPYRHK
LNTAVLKLQENGKISDLKRKWWEERKGGGQCSGEVESQEAKPLTLKNVGGVFWVTVGGV
AVAVVLVFVEMFLHVMKESIKHKAAFWAELSEELKFYMKFKGLVKPVRLKKGDSKSPDDSD
KSEKSEQHEMQGEANGRSYGFLPELIKQPLE
>PbreIR2

MTAFRVVDAEDLAVQEVLRDMVKFQNSLSMPTKLNTSFIQAEAALIYDSVFVFSIGLQTLEQ
SHTLKLPNVSCDREQSWDGGLSLINYINSVEFRGLSGPIEFKEGRRIQFKLDLLKLKQHAIVK
VGEWNPGAGVNVTDRAAFFDPGTMNVTLIVTTILETPYMMMHSSKNYTGNSRFFGFCVDI
LERISRDVGFNYLLDLVPDRKYGVRNPDTGEWNGIVQQLISHELPYVKLKPQSNLTGNARY
EGFCIDLLKCIASMVGFEYRIELVPDGKYGAIDLETGEWNGIVRQLMDKKADLAVGSMTIN
YARESVIDFTKPFMNLGISILFKVPTDKESAFFTFLNPLGFDIWIFVFGAFFMASFTLFTLARFS
PYEWINPTPWKETKFLTNNLSVSNSFWFVTGTLLRQASGVTPQATSTRIVGGIWWFFTLIIISS
YTANLAAFLTVERMITPIENAEDLASQTDILYGTLDSGSTMTFFRDSMIETYKKMWRFMENR
KPSVFVSTYEAGIRRVLEGNYAFLMESTMLDFTVQRDCNLTQIGGLLDSKGYGIATPMGSP
WRDKISLAILEMQEKGEIQMLYDKWWKNTGDICTRNEKGKESKANSLDVDNIGGVFVVLL
CGLAFAVIVAIFEFCYNSKKNALTEKRSPSAPQQSLCSEMGGEFCFALRCRGSRQRPALRRQ
CSKCLSGATYVPTILDIPSHHPQLPRRNNPTNGATQTCQEETIVTVRENSTNAIERYPAREEF
EVT
>PbreIR3
MKPHTYILLFNVVTLFITGTAGAIQNLFGKSLTDKIYEKFFLKTATNDAVVLSDLLIHIFHNHI
TKCVPILLYDEKKYEYFQVIEELVKRLNTSIIHGWVPKSDVKSIGFLKDNQEQTCCNYVLFLN
DIYNYKTFIGKQSVNNVVFVTNSSLWRVNEFLSSEESREFINLLVIAPSAGSKIKRKEPCYVLY
THELYVDGIGSSTTNILASWTNGSLTKKGVKLFSKKIKNGFSGHRFITSVGHQSPFVIKRGPN
EDGEVLWDGIEIRLLKMLAGFYNFTIDIKSIKEDPHKSAAEQVVDSVKSGIANVGLSGIYLTN
ERLDKLDVSYPHSYDCAAFISLTSTALPRYRAIMGPFHWTVWLTLTSVYLLAIFPLTFADRHT
LKHLIKQPQNVENIFWYVFGTFTNSFSLGKDSWANSSKITPRLLMGFYWIFTTIITACYTGSII
AFIMLPNYPNTVDTVKQLLAGRYRIGTLNRGGWEQLFSNSSDILSQKLLKKIELLPDIESGLL
NITKAFFWPYAFLGSRAQLDYIVQTNFTTTNKRSLLHISSECFVPFGVSIVFAKGTIYKDVVD
KGISYIIESGFMKKFEQDIKWDFVRSPTGKLLQANSGTSSKIIYTEDRSLTLDDTQGMFLLLGA
GYLFGLLSLVLENVGGCFGCCGKKRSRENNSTSSNARENSRRCMPKRNNAAMGQADDKE
IEIDRITIQKNKLDALFGEENVHDNNQNY
>PbreIR4
MLPLFRKTILILLTFQKTVLTSPNNSDLIELTKDFLLVEKTSTKVSAYLCWTKDEKISLAKHLN
KHGISFQHLNTFSTPMDEAAIEQLFLVDLRCPEYSTLLWTADQNKLFKQIYRWLVIVGENTT
FSTSLDILVDSKVYLVEKSSISTYSLRSFYKISSRSQEYIINDIATWIADSGFQYYNRLSVTRNRT
NLMGTMFNISYVIVSNDSLNHLWDYRYGHIDGVTKLNYILSHHVTDFMNASRNFLITNTW
GYLNKTTNTYGGLIGHLQSGLAEFSGSPSFFTQDRVAIVDYIGATTPTRAKFIFRDPPLSYVK
NVFKLPFDEMVWYASFLLLGIISFVLYAIVKWEWSEPKFEKVLLQRQDCMRPRYGEVLLMEL
GAVCQQGSETEPRSASGKIATIFVFLTLMAVYTSYSAKIVALLQSTSDSIQTVDDLLNSRIKLG
VELTPYSVYYSKTQTQKTRKAIYEQKIAPPNQEPNFMTAEVGIRKMRDEFFAFHIECGVAYK
LVADTFQESQKCGLREIDYWQIIDPWVTIRKNSSYKELAKLAYRKIHESGIQNRDYNRLYTRK
PVCQSKGSNFISIGLIDCYFPFLIFGIGSAISIGMLFLELLYRHKFSQIIESRRNLKTN
>PbreIR5
MYFPNHLILVLIARICLIESRGNVTSLIDVIIDFISNQKPLAKASINVCWSDSERLYLARQLTNN
FIGFQYNIDVNESRDGLLSAQWFLLDLRCPAYKDLLQNANDKMLFKHPYRWLVIKDDMQ
QIRTDLNIMPDSQFYVINQTSADLYAINSIYKVRKDDDHFVIMDIAKWSSLTKFIYCNYLYPG
RNRTNLMKTNLIFSMVITDPDSLNHLWDYRNRHIDSMTKLNYFFCHHVTDYLNASISFIVR
DTWGYRNKTTKLFSGMIGDLESGLAEIGGSPAVLAQDRMNAIEYIAPTTPTRAKFIFIAPPLS
FVTNILTSPFDVTVWYCTFLLTGVIGIVIYIIVTWEWKNPVYREMLEDQQFAIRANISDVLIMT

LGAICQEGSEAEPKSNSGRIATITVFISVMFLYTFYSANIVALLQSTSESIQTIEDLLNSQIKLGV
DDIAFNRYFLKEQTDRTRKAIYAQKIAPDGQPANFMNATRGIQKVKNEFFAFHVECAVGYK
IIADTFEENEKCRLQEIAYCTEIGNPWMAIKKNSSYKEVAKVA
>PbreIR6
MKVVVMKHTPATTKLPISEDYNVRAIMSDGQIGFAGLEIEILATVSKAMNFRCTLREASNA
DFWGKKESGGKYTGLIGDVVTNHADIALGDLYYTSYVLELMDLTVPYNAECLTFLTPESST
DNSWKTLILPFKLLMWICVMLCLFLCGIIFHYLAQFHCKIQNRQNKPKIDIDKKRKEIITLPIT
SIDQLNPNAKYVLMKQQIKSTRHGKDPNGLYLFSDFFNSILYTYGMILMVSLPRLPTGWSLR
MLTGWYWLYCTLVVVSYRASMTAILANPAPRVTINTLQELVDSKLALGGWGQVNKELFKT
SLDPITQTIGNKFEFVNDSTQSVARVVEGRFALYENIYFLKEASVIHQARLQHTNDDNQSIPL
GNQKNKGDRNMHIMKDCVINMPVSIGLQKNSPIKPRVDKYIRKVFEAGLVRKWLDDVMQ
PTLNSEVPTTLETMKALMSMQKFIGAIVVLSIGYFISLILFGVEVAYFHYAVKKHPNFNKYSR
KIYKKQ
>PbreIR7
MNNVLFPHVVHGFMGKSFSLVTLHNPPWQIIETNDRGGIKCTGLVFDIINELAASLNFTYTL
IVLNGEKNEKKNSSYYGKDISYRMIYSVPDGVVRMIRSKQVFMAAFAYTITDENKAVVNFTI
PITTQPYTLLTATPKELSRALLFISPFTFNTWLCLLAAIGTMGPILYCIHKISPVNAYHGISARG
GLSSISNCTWYIYGALLQQGGLYLPYADSARLLIGSWWLVVLVISTTYCGNLVAFLTFPNND
KPVTTIDELLNQRDTVTWSIAPSTYYEYEIKISNEPKYQALYHGSLQNVGNMDKMLTNIELG
KHVHIDWKLRLQYIMKRRFSLKGTCSLSLGTQDFFDERLGLVVSPDNPYLKRINREIKRLHQ
VGLIEKWLKDYLPKRDKCFKTRSNSNVNNHTVNLDDMQGCFFVLFFGCFISLLLICGERFY
HKYQIKRERNIVQPFVS
>PbreIR8
MLSRNEVDVGAAFLPTFPYISKHIRFSVSLSKAEWVVLMKRPPVSATGSGLLAPFTVEVWLL
ILISLFAVGPIIYLLIILQCRLCHDEGNIIYPLPSCIWFVYGALLKQGSTLSPRTDSSRILFATWWI
FITILTAFYTANLTAFLTLSRFTLPIASVSDIGTKKYSWVSHKGSAIEAALENDVNFKSSLKGSH
WEFLEEDAGNILENWVKRHDYMYIGEKPIVDHLMYRDYLSKINTHIAEAERCTFVITTWTI
TNNLRSFGYSPNFPFVDLFDNILEHLVESGIVMYSLREGLPDTQICPLDLGSKERQLQNTDLI
MTYYIVIGGFIVSTIAFMCELLYIRCNGRHQSSDQRGPDALFVINKNSKGNKLNDNYLFPPP
PPYHAIFKPPFPNSEDTRMRTVNGRDYWVIKTSDGNSKLVPIRTPSAFLFQYAN
>PbreIR9
MIRITDNDTWNHLEDLRYPSVDSLTKLCYGSNRCIMDYCNANVTYYVRTEDFGITDKNGKF
NGIVAGLLSGEGDTSVNALVPTFQRLDAVAYMSVQSPYYIKFVLKKPSLSYIENIYVLVLDKT
VWLVLVIILVVFAFAAFLILNIEAKSDKIGDQEKISVTDVILLALEVLSQQGTSIDPKRISGRILQ
LIFFMAFFFVFIVYSGNVVAMLKAQVVLKSSKELLQTKMTIASQDYNYMHFYVAQDKSELG
KEINRRTGPKGYYSNEVGMENVRKGSFAFQVILNEAYNYMLENFTNSEICSTQEVDGYFEH
LRAYHITQKRSPYKEIFRTGALKVDEHGLRQRNHIRWFVKPHCRTPVANYESVGLIETRMVF
LLVLYGIILSVIFLILENAVYYVRKIRKFRVSMKLKRTTLFKNTTTI
>PbreIR10
MLVAVEYGKRHNMTLEFVVDEENEWGDVYANWTGRGVLGNLVQDKGDVGIGALYSWKE
SFDFFDFSKPVMRTGVTCIAPAPRLASGLSTPFAPFSLKLWIITAIAYILASASMFIILSISDAEN
NQNNPKVQNVEMSFELSGRIFLLQSFQKVPNLEQSRITFGLALILSLLLNTMYSSGLSSTMTI
PKYYGTIHDVNDLVASGMNWGATSTAWISSIESDSREVIKKVVRAFQIINEESLTQLNDRNF
AYAVERLQAGNLAIGSYVTLEGAQRRRLLDSDIYMEFCVLMTRKSSILLPSLNDLVFAVTESG

LVYHWEYQAVVKYMDINTQKAIKESIKTTKHGGTLVKLGLNHVVGAFTIWGAGLLLSIIAFI
IELFTARRIKIALTQP
>PbreIR11
MTIISKKNVSDIISYEFWEMYGLNNRRSILTHTGAWSKFIQKKQQGAFYRDRKNVHGSKLKI
SSEICNTTTGKTLQEKYAFLKIEREINCILLNLIQYIHNFSIGIAQGERLDIISGFMEESLVNITD
VIAQTYPFRKTFILKNPVGGNKNILIKPFSPKVWYMFILLIFITTALLKILLSVLHAFEMVDDK
SWSLVILILLSTICLEGSPIESHFLSFRVFVYTCLLSSMMLYNYYSSNVVSILLTKPAPNIKSLAD
LRKANIKLGVENIPNYYKPESELLTTNDFNAVLNLSEAIVAIKGGDFAYYTEPATRYKLLQER
LTFLEACSLVEIDLVPVAFVTVMARKKMEFKELLKIT
>PbreIR12
MLATFTANLAAFLTVEMMQTPVQSLEQLAKQSRINYTVVESSDTHQYFINMKNAEDTLYR
LWKELTLNASTDDIQYRVWDYPIREQYGHILLAINDSIPVANASEGFRKVNERIDADFAFIH
DSSEIKYEISKNCNLTEVGEVFAEKPYAVAIQQGSLLHDEISKKILELQKNRFFDHLQAKHW
NSSQKGDCDNTDNNEGITLESLGGVFIATLFGLALAMVTLVGEVIYYKRKKNQKISLHPKDI
PTIKTISVKPTIPDTITFGSTFKPAITDKENFRMSHLTLYPRPRSRIN
>PbreIR13
MLPVQFLIGLGFVNFAYCNSFPSLLTVNATLAVIIDKEYLGDNYEFIKTIIEDRINIVKQQKLK
TAGLNVVYSYWTNIYIKKEISVILTVASCKDTWRLHKMADAENILHLAICETDCPRLPVDKA
FTIPMTVRGEELPQIIFDLRSRKAYDWKSVILLYDDSLGRDFTTRVLSSLTVDTTELSTGSTSVS
LIKLDDYNTGLDKSSIKSTLSTFTSQISRSSFIVIVSMDLATYILQAAQSLELMDITSQWLYVVS
DYKLDNDTTSEILSNLKEGNNVAFLNNASTSSTSC
>PbreIR14
MNYEWYKATSTRIVGGIWWFFTLIIISSYTANLAAFLTVERMITPIENAQDLAEQTEIAYGTLE
GGSTMTFFRDSKIGIYQKMWRFMESKRPAVFVSTYDEGVQRVLNGNYAFLMESTMLDYAV
QRDCNLTQIGGLLDSKGYGIATPKGSPWRDKISLAILELQEKGVIQILYDKWWKNTGDVCN
RDDKSKESKANALGVENIANRSVAVRLKTDNVLLDIYNTIVMPAVAAKTRSHRIHSLYIIIIFV
LAVWYVQALIYYITNKMSDFY
>PbreIR15
MPFMTLGISILYSKAAKEPPDLLSFMQPLSLDIWLFMATAYLIISIIIYFAARLTPREWESPHPC
DDNPVELENIWTIKNSFWLTLGSIMAQGCDILPKGLSTRMITSMWWFFSLIITSSYTANLAAF
LTNERMGPTIESVEDLAKQSKIKYGCLEGGATAAFFRGSNFSTYQRIWRTMVSAEPSVFHKT
NKDGVKSIKNSKRMYAFFMESSTIEY
>PbreIR16
MFIYVAYSANIVAMLQSQVELKSTKELLESKLTVGAEDDVYVQAYISMNTHKDIYQKKIDA
GGYYLLEDGMSKLRKGYFALHVDLCTAYNYIRRTFTAYEICSLQEIDGLFTNLKAYGVVPRD
SPYKEIFKTGLIRIDEYGLRHRNFVRHFVKPICSGQVASFGSIGLVDTQMPFMLIIYGYLLAIFV
LLIEKIISKLIH
>PbreIR17
MYAFFMESSTIEYERERDCDLIQVGGWLDTKGYGIAMPINSPYRTAINEAILRLQESGTLRRL
KEKWWRQMHGGGQCNETESSSDDSAAELGIDHVGGVFVVLTGGVILAFLIAAFEFLWNV
RKISVQGQMTFTQALAQELKFALRVSDSKKAVATKRTPARDAQT
>PbreIR18
MAKGFAALVASMNWKSFTVLYETTESLAKLRDVLKINVPRELPMIFKQIHPDEDYRVVFKQI
KNYTENTIIIDCELDRVIPILQQAKEVGMLEFHNKYFLANLDAHTLDFSDLSTTANITTISLL

NTTLPILRHATKDWSDGRKSR
>PbreIR19
MYSANLTSLLAKPGREKSINNLIQLEEAIKYEDFQLFVEKHSSTHSLLENGTGIYGRLWELM
NTVQSRYLVNSVEEGVKLVKDFSDVAVIAGRETLFFDIQRFGPINFHLSEKINTAYSAIAFQLG
CPYVENVNKILMAIFEA
>PbreIR20
MIENDVHWGATEDAWILSIEKSESPTHKKIVNLFVIENEDELAERNTDDRFAFAIERLQGGN
FAVGDYVSEDGAKIRRILDEDLYFDYMAFYLRKSSILLPYLDDIVLRVAQAGLLDVWEYNVF
NS

SNMP
>PbreSNMP1
MVILGDIFSDSQWPKRRDNIRNLVCCLCFPKLSALLALYNRGYLRKFDVRNMQFYARLALA
SGIILTCITIFGWVAFPNLIRSKIKSSVSLKPGNEIRDMYMKIPFPLTFKIYVFNITNPDEIAMGQ
TPIVQEVGPFMFEEWRSKVFVSEDEEEDTMTFFSKETFIFKPGTLTGNEVLTIPHPLIVAILLTV
IRDKPSAVNLINKAMNSIFKKPKTPFLTATFRDIFFDGVIVNCSVTDFAGKAVCTQLKTEGKD
FEYIEENVFKFSLLGAKNASATNKFKVKRGVKDYKETGRVLEYNDKPQLDVWSTEECNQY
MGTEGTIFPALIPHGADIPSFAAMLCRSMKAKFTGNTKYNGIPVSRYTLDFGDQSNNPEDK
CYCPSPDECMKKGALDLRKCSGPIVATMPHFYNSDPSFVAGVKGLNPEKEKHELYLLFETM
TGSPLAARNRLQFSIPFVPMPKVDVMKNVPDTLVPMFWVEESIDLNKTLTKPIKTLYVTMK
TVRIISWLILLGTLGGLGYAGFLYYKRGREIQITPVKSVKKDNGISTVMSKGDDGIKGGVSNP
GVTDNDIDKY
>PbreSNMP2
MYKHCPIIVAVNGFLILILGIVCTWYGFPTLVEHQINQNIKLREGSQAFDMWKKQPFPLFYKI
YFFDVQNTEEVMLGKKPILKERGPYVYDLHIERQIIEIDEDADTIKYHLKKHFVFNATASGC
CSDDDVVTIVNAALVGTVLKVNSMLPGLIDMVIKGMPYIYPNITDLFLRVKVKDILFDGITL
YCSAPEIASICSIMKGARPSTMKIAANDKDLIFSMMSAYNDTLQGPLTMTRGIKNTTRGDLV
NYRFHKMLDDWEKDPPYCNRLNGSDSGLYFPMKKPVPIMYTFVPEICRSVASFFERKMTKS
GLEVYKYASTEESLSTAGPYQCFCAPDENDTYLCPPKGVINLEPCLMAPVLMSNPHFYLGD
KKLLDYAGSLKPVKEKHESYLILEPRTATPLEGAKRTQMNIEIKQYDNFPLLENVTGGIFPIL
WLEEGVELPENLVHLLQTSFDQIALLDTIQWILIATGIALMIISFVLVMYQEKLMCFSTSGSVK
VTEPGLFTTRTSNLNFNTSVVYPKHDNPAFVNNEDLDRDKRHYSNNRLS
>PbreSNMP3
MFAGSQLFTPTQDGIEFSFFKSKDNVQQDFQILAGIEEIYDVGQILSWNHAKFMNVWVNN
DSICNEIRGTDGTLFQPFNNQESSFDVFYNDICRIFNFQFDSEAEFIEINGYR
>PbreSNMP4
MRLQLESPPSHVLKYASSSQESISPRVLTSLFPADILYPPSLAFCLCEKSANVILSLSLVNVLEFV
ICSFTRLQKILCAGFIERTQKS
